# The Influence of the Processing Parameters on the Laser-Ablation of Stainless Steel and Brass during the Engraving by Nanosecond Fiber Laser

**DOI:** 10.3390/nano12020232

**Published:** 2022-01-11

**Authors:** Luka Hribar, Peter Gregorčič, Matej Senegačnik, Matija Jezeršek

**Affiliations:** Faculty of Mechanical Engineering, University of Ljubljana, Aškerčeva 6, 1000 Ljubljana, Slovenia; luka.hribar@fs.uni-lj.si (L.H.); matej.senegacnik@fs.uni-lj.si (M.S.); matija.jezersek@fs.uni-lj.si (M.J.)

**Keywords:** laser ablation, engraving, fiber laser, material removal rate, laser-material interaction

## Abstract

In this paper, we investigate the influence of the following parameters: pulse duration, pulse repetition rate, line-to-line and pulse-to-pulse overlaps, and scanning strategy on the ablation of AISI 316L steel and CuZn37 brass with a nanosecond, 1064-nm, Yb fiber laser. The results show that the material removal rate (*MRR*) increases monotonically with pulse duration up to the characteristic repetition rate (*f*_0_) where pulse energy and average power are maximal. The maximum *MRR* is reached at a repetition rate that is equal or slightly higher as *f*_0_. The exact value depends on the correlation between the fluence of the laser pulses and the pulse repetition rate, as well as on the material properties of the sample. The results show that shielding of the laser beam by plasma and ejected material plays an important role in reducing the *MRR*. The surface roughness is mainly influenced by the line-to-line and the pulse-to-pulse overlaps, where larger overlap leads to lower roughness. Process optimization indicates that while operating with laser processing parameters resulting in the highest *MRR*, the best ratio between the *MRR* and surface roughness appears at ~50% overlap of the laser pulses, regardless of the material being processed.

## 1. Introduction

Laser ablation, a process in which material is removed layer by layer by systematic guidance of a laser beam over a sample, also known as laser engraving or laser milling, has been established in recent decades as an alternative to conventional methods of material removal in a wide range of industrial applications [1,2,3,4]. In addition to many outstanding properties of laser milling compared to conventional milling techniques, the recently increased interest of the industry [5,6,7,8,9] has been mainly influenced by the development of new laser sources which are more efficient and adaptable in terms of pulse energies and temporal shapes.

In the context of industrial applicability, the optimization of laser engraving is mainly related to the tradeoff between the material removal rate (*MRR*) and the quality of the treated surface. It is desirable to increase the *MRR* while maintaining a high quality of the surface. High quality laser engraving depends on minimization of unwanted side effects, such as the heat-affected zone (HAZ), burrs, microcracks, and remolten material. It is well-known that these side effects increase by increased pulse durations [10,11,12,13,14,15]. Thus, ultrashort femto- or picosecond laser pulses are usually used to maximize the quality of the surfaces after laser treatment [16,17]. However, ultrashort laser systems are significantly less interesting for industrial use compared to nanosecond ones, considering the high cost and more complex operating conditions due to the high intensity of the laser light (>10^13^ W/cm^2^) [18]. The additional advantage of nanosecond laser sources is also reflected in the material removal rate, as they achieve significantly higher values at the same average laser power as ultrashort lasers due to different ablation mechanisms [13,19,20]. In recent years, intensive research [21,22,23,24,25] has therefore been conducted into how the processing parameters of nanosecond laser sources affect the ablation result, with the aim of achieving a comparable quality of the treated surface as after treatment with ultrashort laser systems, which would enable the expansion of this technology to new branches of industry.

Many studies confirm that the outcome of laser ablation can be controlled to some extent by manipulating key parameters, which can be divided into three categories. The first one is defined by the laser system and encompasses the pulse peak power (*P*_max_) [26], waveform (WF) [2], pulse duration (*t*_p_) [1,2,10,27,28], average power of laser light (*P*_avg_) [4,11,27,28,29,30], pulse repetition rate (*f*) [1,11,27,28,29,30,31,32,33,34], scanning speed (*v*) [26,27,28,29,30,31,32,33,34,35], and different scanning strategies [1,27,29,33,35,36]. However, these parameters should be optimized regarding the influential physical properties of the processed material, e.g., absorption coefficient (*α*), thermal conductivity (*κ*), specific heat capacity (*c*_p_), latent heat of melting (*L*_m_), and latent heat of vaporization (*L*_v_) [2,4,11,32,34,36,37,38]. The third category of the processing parameters includes some environmental conditions, such as processing atmosphere and ambient temperature [3,10] that also cannot be neglected.

Numerous studies, both scientific and industrial, have been carried out to investigate the correlations between surface quality and *MRR*. Researchers have studied the effects of different parameters when processing metallic [1,2,4,10,11,26,27,29,31,32,33,36] and non-metallic [4,15,28,34,35] materials. However, most of the investigations have so far been performed with classical Q-switched diode-pumped Nd:YAG [4,26,28,29,32] and Nd:YVO_4_ [27] solid-state lasers. The new alternative to these conventional laser systems are much less researched pulsed Yb fiber lasers based on a master oscillator power amplifier (MOPA) architecture [1,2,37], which exhibit numerous superior characteristics, such as the flexibility in the control of a wide range of pulse durations and/or waveforms [37,39,40], allowing different processing operations to be performed with the same laser source. In this regard, their indispensability is already evident in the industrial applications of marking [41,42,43], removal of thin films (production of solar panels) [44,45], surface functionalization [46,47], engraving [48], drilling [49,50,51], cutting [52,53,54,55] and rapid prototyping [56]. They are also of general interest for use in many cases of microproduction [57,58,59], as they allow the formation of complex three-dimensional (3D) shapes in various industrially interesting materials, e.g., polymers, metals, ceramics, and organic materials [2]. Thus, fiber lasers with MOPA architecture allow investigations of how pulse duration and overlapping of different pulses at high repetition rates influence the ejection of the remolten material and the plasma formation, e.g., feeding the plasma plume by consecutive pulses and plasma shielding.

Studies [2,4,11,32,34,36,37,38] show that despite some general mechanisms, the influence and, consequently, the optimization of processing parameters is also strongly related to the material being processed. Considering that laser ablation is a process that is very interesting from an industrial point of view due to its applicability, it is not surprising that the research has mainly been carried out on materials that are most widely used, i.e., aluminum and steel. There are far fewer studies on less common industrial materials, so further research in this area is still needed.

The main objective of this study is to fill the gap in the investigation of the influence of process parameters on the ablation of CuZn37 brass using a nanosecond, 1064-nm, Yb fiber laser based on a MOPA architecture. In order to achieve this goal, the influence of the following parameters: pulse duration, pulse repetition rate, line-to-line overlap and pulse-to-pulse overlap, and scanning strategy, on the material ablation rate (*MRR*) and the quality of the treated surface characterized by surface roughness *S*_a_ was systematically investigated. Additionally, we examined the effects of the use of different pulse fluences and different number of scanning transitions (*N*) on the process outcome. To improve the understanding of the light-matter interaction at high repetition rates (>10 kHz), we employed imaging by a high-speed camera to observe the processes leading to the macroscopic transformation of the surface, i.e., melting and vaporization of the material and plasma formation. The reason why brass was chosen as the material of study is that it is used for sliding elements in mechanical systems and therefore the possibility of laser structuring of the surface is very interesting, especially to improve its tribological properties. We have also carried out the same investigation on AISI 316L steel for comparison.

## 2. Materials and Methods

### 2.1. Laser Processing

**Figure 1:**
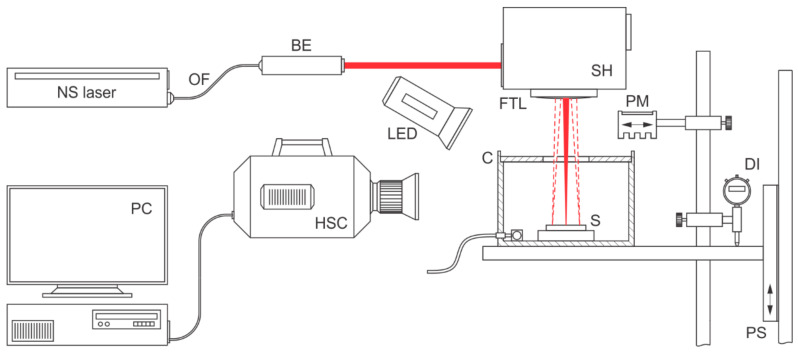
Experimental setup.

The experiments were conducted using the experimental laser processing system, presented in Figure 1. A 20 W pulsed Yb fiber laser (G4 type, SP-020P-A-HS-S-A-Y, SPI Lasers, Hughington, UK) with fundamental wavelength λ = 1064 nm, a variable pulse repetition rates in the range of 1 kHz to 1 MHz, a pulse energy up to 0.6 mJ and a peak power up to 10 kW was used. Based on MOPA architecture, the laser pulses of this laser system are generated by the semiconductor seed laser that determines its characteristics, while the desired output power is achieved by two-stage optical amplification. This operation mode allows the formation of different preprogramed waveforms, each one optimized in terms of pulse energy and peak power at its specific pulse repetition rate referred to as *f*_0_. The detailed characteristics are presented in Section S1 of the Supplementary Materials. Waveforms with pulse durations between 70 ns and 240 ns were used in this research. Their relevant specifications are summarized in Table S1 of the Supplementary Materials.

A guiding optical fiber (OF) delivers the laser beam with the beam quality *M*^2^ = 1.3. We used two different beam expanders (BE; with output beam diameters *D* of 5 mm and 7.5 mm) and the spot size was calculated as 4*M*^2^*λf*/*πD*. The spot sizes that equal 57 for *D =* 5 mm and 38 µm for *D* = 7.5 mm provide two different sets of pulse fluences. Other components of the experimental setup include a scanning head (SH) with an F-theta focusing lens with a focal length of 163 mm, material samples (S), a processing chamber (C) filled either with an air or an inert gas (argon), a power meter (PM), a vertical positioning stage (PS), and a digimatic indicator (DI). In addition, a high-speed camera (HSC) and two LED illumination sources (LED) were implemented in the system when light-matter interaction processes were studied.

The material was ablated layer by layer by periodical guidance of the laser beam across the workpiece surface along the selected pattern, with the focus of the optical system fixed on the original surface of the workpiece. We used a simple pattern consisting of parallel lines in an arbitrary direction that were spaced for a constant distance Δ*y* (Figure S3). The experiments were conducted with different scanning strategies (Figure S8). The latter refers to the periodicity of the scanning transition sequence used for material processing, where the scanning transition represents a single passage of the laser beam over the surface of the workpiece along parallel lines in any direction. More details on the experimental design are provided in Sections S2 and S5 of the Supplementary Materials.

### 2.2. Material

All experiments were conducted by ablating square areas 3 × 3 mm^2^ in size on the samples with dimensions 40 × 40 × 1.5 mm^3^ (see also Figure S6) made of AISI 316L stainless steel and CuZn37 brass. Their physical properties are given in Table 1, while their detailed chemical composition is presented in Section S3 of the Supplementary Materials.

To ensure high repeatability of the experiments, all samples were cut from a single piece of rolled metal sheet. Prior the processing they were cleaned, first with a light degreaser then in an ultrasonic bath with distilled water and finally they were also rinsed by ethanol. The samples were unmodified before processing, with the *S*_a_ of steel and brass equaled 0.175 ± 0.02 µm and 0.200 ± 0.05 µm, while their *S*_z_ were 6.225 ± 0.35 µm and 4.550 ± 0.35 µm, respectively.

### 2.3. Characterization of Ablation Outcomes

The results were evaluated by using two quantitative variables, material removal rate (*MRR*) and roughness of the ablated surfaces (*S*_a_). The *MRR* we define by the amount of material volume *V* removed in a unit of time *t* as:

(1) $$MRR=\frac{V}{t}.$$

The characteristics of the ablated volumes were determined based on the reference measurements conducted by a 3D optical microscope (Alicona G4 Infinite-Focus, made by Alicona, Raaba/Graz, Österreich). Their shapes are not prismatic, although the external dimensions of all scanning transitions are identical (3 mm × 3 mm). The relief at the bottom of the pockets is quite rough and wavy. The cross section remains square-shaped, but its dimensions decrease with depth. Although the pocket walls are not vertical, we have found that their inclination does not change significantly with depth. Thus, we decided to use a right 4-sided truncated pyramid as best approximation of the shape of the ablated volumes. Afterwards, an optical microscope with a displacement detection module (digital positioner, type 9598 S.N. I7500 and digital display microcode II, type 3 M, both made by Boeckeler instruments, Tucson, Arizona) was used to measure the depths of the pockets needed to calculate their volumes and, consequently, the *MRR*. Further details are available in Section S4 of the Supplementary Materials.

Surface roughness *S*_a_ is defined as the mean arithmetic deviation of all measured points of a two-dimensional surface profile:

(2) $$S_{a}=\frac{1}{m\;n}\sum_{i=1,j=1}^{m\;n}|y_{i,j}|,$$

where |*y*_i,j_| is the absolute value of the deviation from the mean at the corresponding point. We obtained it by employing the 3D optical microscope (Alicona G4 Infinite-Focus). The sampling range was approximately 2 mm × 2 mm in size and a high pass filter with a cutoff wavelength of 140 µm was applied to eliminate the low frequency waviness of the surface.

### 2.4. Plasma Characterization

The plasma plume, which is generated during laser ablation, defocuses the laser light on its way to the workpiece and absorbs part of the pulse energy. This contributes to reduction of the ablation of the material itself and it has a negative effect on the energy efficiency. The phenomenon is mainly related to the intensity of the plasma, i.e., the density of the medium traversed by the laser beam and its dimension. To study these shielding phenomena, we used a high-speed camera (Photron, Tokyo, Japan, model Fastcam SA-Z, type 2100K-M-64GB) and a macro lens (Sigma, Aizu, Japan, model APO MACRO 180 mm F2.8 DG HSM). The camera layout was perpendicular to the angle of incidence of the laser beam. Additionally, two Ryoyu LED sources were used, each generating 50k lumens of light at full power, to provide enough light during short exposure time of the camera (8.4 µs).

The detection system parameters for the observation of light-matter interaction at high pulse repetition rates during laser ablation, which were adjusted according to the time frame of the studied process and the dimensions of the region of interest, are presented in Table 2.

## 3. Results and Discussion

The influential parameters were divided into (*i*) laser processing parameters, i.e., pulse repetition rate *f* and pulse duration *t*_p_; and (*ii*) scanner parameters, i.e., scanning speed *v* and scanning line separation Δ*y* that defines the line-to-line and the pulse-to-pulse overlaps, laser spot size *d*_f_ and scanning strategy.

The line-to-line overlap *η*_|−|_ is defined as:

(3) $$\eta_{\left|-\right|}=\left(1-\frac{\Delta y}{d_{f}}\right),$$

while the pulse-to-pulse overlap *η*_p−p_ can be calculated as:

(4) $$\eta_{p-p}=\left(1-\frac{\Delta x}{d_{f}}\right).$$

In Equation (4), the spacing between the individual pulses, Δ*x*, depends on the scanning speed and the pulse repetition rate as:

(5) $$\Delta x=\frac{v}{f}.$$

In the following subsections, we firstly present, how the laser processing parameters influence on the *MRR* and surface roughness *S*_a_. Here, we also discuss the influence of the plasma shielding. Then, we show the influence of the scanner parameters on *MRR* and mean maximum surface depth (*S*_z_).

### 3.1. Influence of Laser Processing Parameters on MRR

**Figure 2:**
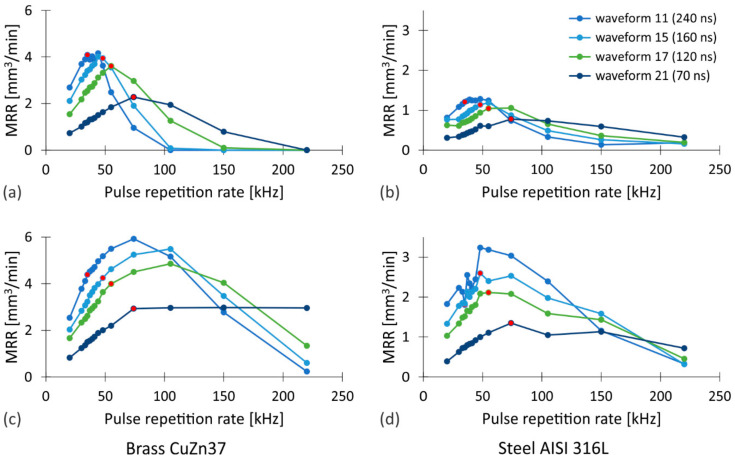
The evolution of *MRR* as a function of the pulse repetition rate and the pulse duration when processing (**a**,**c**) brass CuZn37 and (**b**,**d**) steel AISI 316L using two sets of pulse fluences of (**a**,**b**) 20 J/cm^2^ and (**c**,**d**) 45 J/cm^2^. The repetition rates corresponding to the *f*_0_ of the selected waveforms are marked with the red dots.

The dependence of *MRR* on the pulse repetition rate, pulse duration, material type and pulse fluence is presented in Figure 2. To determine the influence of pulse repetition rate and pulse duration on laser ablation, we used seven different pulse durations (70–240 ns) corresponding to the waveforms that are revealed in Table S1 (Supplementary Materials) at repetition rates in the range 20–220 kHz. Experiments were also conducted with two different sets of pulse fluences, i.e., higher (45 J/cm^2^) and lower (20 J/cm^2^), that were achieved by two different beam diameters on the F-theta lens. Fluence is calculated as a pulse energy per area within the beam waist radius (*F* = *E*_p_/π*w*_0_^2^). Thus, the peak fluence equals twice this value [63]. When we increased the pulse repetition rate, we also increased the scanning velocity to keep Δ*x* constant as it is determined by the Equation (5). Thus, both the line-to-line and the pulse-to-pulse overlaps are equal to 50% for all measurements in Figure 2. Further details regarding the experiments are available in Sections S1 and S5.1 of the Supplementary Materials.

As can be seen from Figure 2, the *MRR* increases with increasing pulse duration up to a certain pulse repetition rate, after which the trend reverses. In contrast, the maximum achievable material removal rate *MRR*_max_ for a given waveform increases continuously with the pulse duration for both materials studied. This can be associated with the relationship between the pulse duration and the maximum fluence, which originates from the specifications of the laser system used. Up to a certain pulse repetition rate *f*_0_, the average power of the laser increases linearly with the repetition rate, while the pulse energy remains constant (see Figure S1). When the repetition rate reaches *f*_0_, both the average power and the pulse energy are at their maximum values. For pulse repetition rates above *f*_0_, the situation reverses. The average power remains constant while the pulse energy decreases as:

(6) $$E_{p}=\frac{P_{\mathrm{avg}}}{f}.$$

Due to laser characteristics, each waveform has different energy maxima while their maximum average power is identical. As a result, each waveform has its own specific *f*_0_, while the general characteristics of the system remain unchanged regardless of the waveform selected. That being the case the maximum energy and thus the maximum fluence of a single laser pulse depends on the selected waveform, namely the longer the pulse, the higher its maximum achievable fluence. The described laser characteristics are also the reason for the observed shift in the relationship between *MRR* and pulse duration at high pulse repetition rates. As mentioned earlier, at pulse repetition rates above the *f*_0_ of the specific waveform, the average power of the system remains constant. When the pulse repetition rate is higher than the highest *f*_0_ of all relevant waveforms, the energy of each pulse is the same (and equals *P*/*f*) regardless of its duration (Figure S4 of the Supplementary Materials). Therefore, for longer pulses, where the same energy is distributed over a longer time interval, the intensity is lower and, consequently, the *MRR* is also lower. Further details on the dependence of the pulse fluence on the pulse repetition rate are presented in Figure S4 (Supplementary Materials).

The influence of the pulse repetition rate is much more complex due to the interdependence of the processing parameters. The *MRR* increases linearly to the specific repetition rate which corresponds to *f*_0_ of the selected waveform (marked with the red dots in Figure 2). This is consistent with the characteristics of a laser source whose average output power is linearly dependent on the pulse repetition rate up to the *f*_0_ (Figure S1 of the Supplementary Materials), while the pulse energy for these repetition rates remains constant. At higher repetition rates, the trend reverses and the maximum *MRR* is achieved at a pulse repetition rate that is higher or at most equal to the *f*_0_. To a certain extent, this can also be explained by the dependence of the pulse energy on the repetition rate. While the average power remains constant above the *f*_0_ the pulse energy decreases, and consequently lowers the *MRR*. The results obtained with lower pulse fluences that are shown in Figure 2a,b confirm this, as the maximum *MRR* occurs at a pulse repetition rate roughly corresponding to the *f*_0_ of each specific waveform for both materials studied. On the other hand, the use of higher fluences, i.e., higher energy density on the workpiece, shifts the maximum *MRR* to slightly higher repetition rates, which confirms that the material removal does not depend solely on the already mentioned laser characteristics.

**Figure 3:**
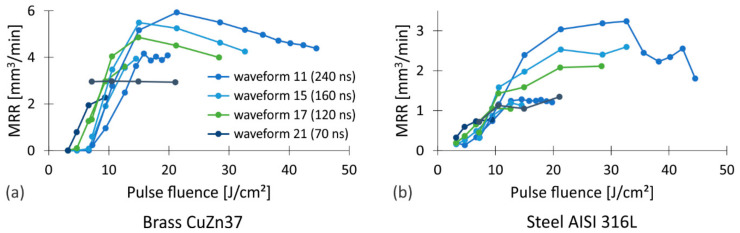
The evolution of the *MRR* as a function of the pulse fluence when processing (**a**) brass CuZn37 and (**b**) steel AISI 316L.

**Figure 4:**
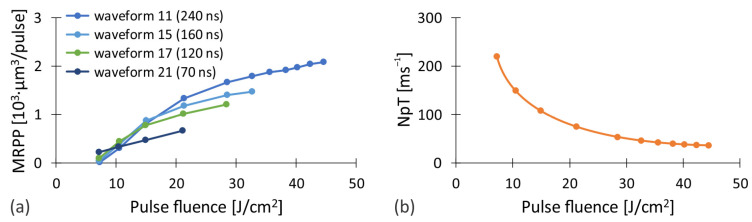
(**a**) *MRPP* and (**b**) *NpT* as a function of pulse fluence when processing brass CuZn37.

To clarify the above-mentioned findings, the results from Figure 2 are additionally presented as a function of the pulse fluence on the workpiece surface. This ensures the comparability of the results regardless of the system configuration used. The combined experimental data obtained with both sets of pulse fluences are shown in Figure 3. Good agreement can be observed in the area where the fluences overlap. This suggests that the pulse fluence significantly affects the *MRR*. It increases with increasing fluence up to a certain value, after which the trend reverses. While the growth rate and location of the maximum varies with both the workpiece material and the waveform of the pulses, the determination of the exact value of *MRR*_max_ for a particular case depends mainly on the correlation between the fluence of the laser pulses and the pulse repetition rate. To clarify this interdependence, we show the results for brass from Figure 3 using two different contributions, the material removed per laser pulse (*MRPP*) presented in Figure 4a and the number of laser pulses per time unit (*NpT*) shown in Figure 4b.

Pulse repetition rate affects the number of laser pulses that are generated in a unit of time, the pulse energy and, consequently, the pulse fluence. At a constant average power (i.e., at *f* > *f*_0_), the pulse fluence decreases by the repetition rate as *P*_avg_/(π*w*_0_^2^*f*). Thus, lower pulse fluence means higher number of pulses per unit of time (Figure 4b). Higher pulse repetition rate also results in a shorter time interval between consecutive laser pulses. This increases the possibility of interaction of the laser pulse within the cloud of the ablated material that appears in different physical states above the processed area. The interaction of the laser light and the ablated material results in partial absorption and distortion of the laser pulse, which negatively affects *MRPP*. On the contrary, the time window for heat dissipation from the processing area is shortened by increasing the pulse repetition rate. As a result, the temperature of the workpiece locally increases. Therefore, less energy needs to be provided for the ablation of the material and also the absorbance of the surface increases [64], which results in the increased *MRPP*.

The increase in the fluence of laser pulses (by decreasing the pulse repetition rate) and the consequent increase in the input energy per unit area, on the other hand, enhances the amount of ablated material per individual pulse (Figure 4a). While the dependence is linear at relatively low fluences, at higher fluences the curve slowly flattens out. The reason for this is most likely related to the dimension of the plasma plume and the intensity of other accompanying ablation processes, such as injections of the molten material and generation of a vapor cloud. To confirm this, we analyzed the plasma expansion during the processing.

Same graph as shown in Figure 4, associated with a study conducted on stainless steel, is available in Section S6.1 of the Supplementary Materials. The trend of the results is consistent with the findings presented in the discussed study of brass.

### 3.2. Evaluation of Plasma Shielding

We performed the plasma characterization at 14 different pulse repetition rates (within the range 20–220 kHz) that are relevant for the study of the laser processing parameters and their influence on the ablation outcome (Section 3.1 and Section 3.2). The values of other influencing parameters we selected in accordance with the previous findings and are available in Section S5.4 of the Supplementary Materials.

**Figure 5:**
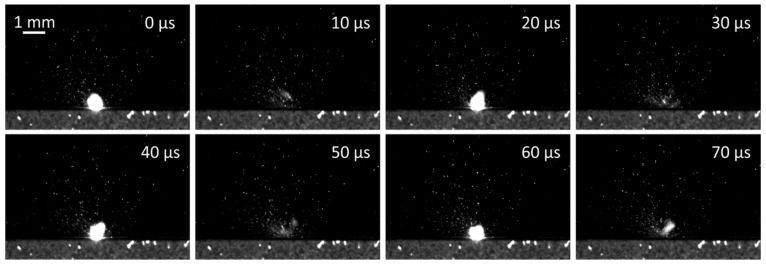
Laser ablation of stainless steel AISI 316L using pulse duration *t*_p_ = 240 ns and pulse repetition rate *f* = 48 kHz.

Figure 5 shows a typical sequence of the pre-processed images that visualize the interaction between the laser light and the stainless steel surface. In this case, the pulse repetition rate equals 48 kHz and the laser pulses of 240 ns are led by a scanning speed of 1440 mm/s over the surface in the direction of the camera axis. As we used the frame rate of 100 kfps (with exposure time of 8.5 µs), the plasma plume is visible in each second image in Figure 5 and is followed by the ejection of molten and evaporated material. Between two pulses, the laser beam is moved for 30 µm (away from the camera), while the plasma is approximately 0.6 mm wide and the ejected particles appear in the region of several mm.

**Figure 6:**
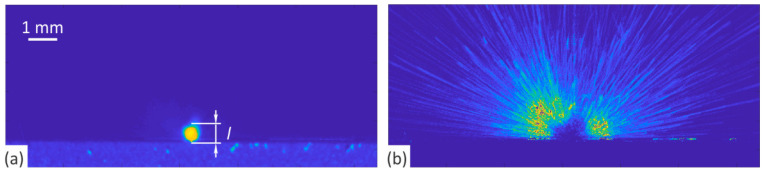
Results of the data processing that allowed (**a**) evaluation of the plasma cloud and (**b**) characterization of material ejections.

Figure 6 shows an example of a processed sequence. Plasma length *l* was determined for each set of influential parameters based on the integration of 15 consecutive images. Intensity filter with threshold of 0.02 of a maximal value was used to eliminate the noise from the results. Typical results are presented in Figure 6a. One can observe that the ejections of molten and evaporated material from Figure 5 are no longer visible in the integrated image. This is because the location of the cloud is fixed, while the ejected particles move radially away from the light-matter interaction site. Increasing the number of integrated images, therefore, seemingly reduces the intensity and thus the visibility of the ejected material compared to the plasma cloud. While this approach facilitates plasma analysis, a substantial amount of information is lost. The ejected material blocking the path of the laser beam has a similar effect on the energy efficiency of the process as the generated plasma. Although the probability of interaction between laser light and particles in the plasma cloud is significantly higher than with those leaving the breakdown area in a lower energy (solid, liquid, and gaseous) state, the expansion rate of ionized material is significantly higher than the ejection rate of molten matter. The plasma lifetime (a few µs) is thus incomparably shorter than the time the molten material remains in the area of potential interaction with the laser light (a few ms). Consequently, the plasma only affects the pulse that generates it, while the ejected particles may shield several successive pulses, depending on the repetition rate and the scanning speed of the system.

With this in mind, the results were also presented in such a way as to allow characterization of the spatial distribution of the material ejections. Similar to the processing of the results to determine the height of the generated plasma (Figure 6a), an intensity filter with a threshold of 0.02 of the maximum value was used to eliminate noise, while the number of integrated consecutive images was increased to 300, corresponding to an integration time of about 3 ms. To improve the intensity resolution of the material ejections, the plasma clouds were filtered out from each image by applying a filter that allowed the elimination of objects with >35 pixels from a binary image. To avoid the occurrence of artifacts that could arise as a result of light reflections at the edge of the samples, the substrate was also filtered out using a spatial filter. Finally, an additional two-stage intensity filter (range of 5 sigma around the mean intensity value) was applied. The typical results are shown in Figure 6b. The uniform spatial distribution of the ejections clearly confirms all the concerns raised, especially considering the high repetition rates typical of the laser engraving process.

**Figure 7:**
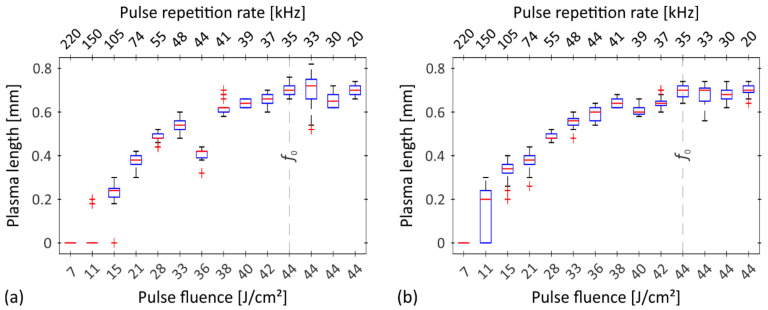
Plasma length as a function of pulse fluence when processing (**a**) brass CuZn37 and (**b**) steel AISI 316L. Outliers are marked with the red plus signs.

Figure 7 shows the results of the plasma length *l* (determined as shown by the label in Figure 6a) for both studied materials. To ensure low statistical risk, the analysis was carried out on a 20 of different image sequences (each sequence is made by averaging 15 consecutive images), and the results are presented as boxplots with an outlier threshold of approximately ±2.7 standard deviations. As expected, the plasma cloud increases with increasing pulse fluence, thereby extending the path of the laser beam through the plasma. In terms of the pulse repetition rate, this means that the plasma length increases with decreasing repetition rate at constant average power (i.e., down to *f*_0_). A further reduction (below *f*_0_) of the pulse repetition rate has no more influence on the plasma dimensions, as at these repetition rates in case of our laser source the average power decreases and the pulse energy (and, consequently, the pulse fluence) remains constant (i.e., 44 J/cm^2^; see the last four numbers in bottom abscissas in Figure 7).

In the case of the nanosecond pulses, plasma generation occurs in the initial phase of the pulse. Therefore, the laser light is subjected to plasma shielding for most of the pulse duration, hence defocusing of the laser beam and energy accumulation in the plasma itself. Consequently, the pulse energy that participates in the ablation of the material is reduced and this has a negative effect on the *MRR*. The extent of reduction is directly related to the size of the plasma cloud, which in turn is related to the pulse fluence.

As already mentioned, there is a significant correlation between the fluence of the laser pulses and the pulse repetition rate. Their augmentation effects several other processing parameters as well as the *MRR* itself, which rises to some extent with an increase in both pulse fluence and pulse repetition rate. The rise is reasonable to the point where the negative effects on the *MRR* outweigh the positive ones and the trend reverses (Figure 2c,d).

### 3.3. Influence of Laser Processing Parameters on S_a_

Based on the findings obtained by the analysis of the influence of laser processing parameters on *MRR*, we reasonably limited the range of variable parameters for studying the surface quality. Surface roughness *S*_a_ we evaluated only using a laser system configuration with a maximum fluence of 45 J/cm^2^ (i.e., with BE output diameter of 7.5 mm). The influence of pulse repetition rate we determined on the basis of measurements at all previously used repetition rates at waveform 11 (*t*_p_ = 240 ns), which resulted in the highest material removal rates. On the other hand, the influence of pulse duration we studied by using seven different waveforms at repetition rates that also proved to be optimal for each particular combination of pulse length and material studied. Further details regarding the experiments are available in Sections S1 and S5 of the Supplementary Materials.

**Figure 8:**
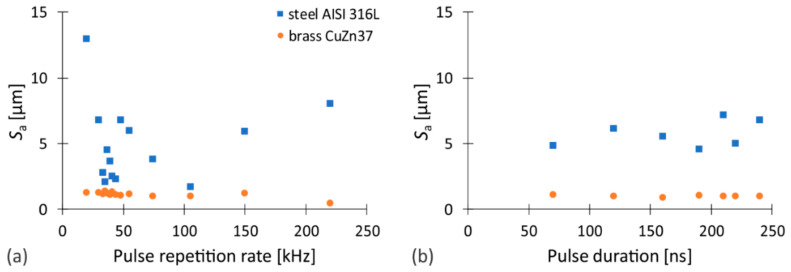
The evolution of the surface roughness as a function of (**a**) the pulse repetition rate and (**b**) the pulse duration.

As is visible from Figure 8a, the surface quality does not change significantly with variation of the pulse repetition rate while ablating both studied materials, namely stainless steel and brass. This contradicts the findings of Williams et al. [1]. The main reason for the observed disagreements is the aforementioned fixation of the degree of overlap of both the laser pulses and the scanning traces, the effects of which are examined in Section 3.4. The *S*_a_ slightly deviates only at a pulse repetition rate significantly lower than *f*_0_ while processing stainless steel. The latter is consistent with the energy efficiency of the ablation (Figure S10), which is the highest when using this combination of experimental parameters (WF 11; *f* = 10 kHz). Thus, with a constant number of scanning transitions, the maximum depth of the ablated volume is obtained and this has a direct effect on the *S*_a_, as it increases with depth when processing AISI 316L steel (see Section S6.3). Additionally, the influence of the average laser power, which increases up to *f*_0_, should also not be neglected. The lower the pulse repetition rate compared to *f*_0_, the greater the temperature fluctuations of the workpiece during ablation, since despite the energy of the pulses remains constant, the time between pulses increases, causing the workpiece temperature to drop before the next laser pulse.

On the other hand, the surface roughness spectrum of brass is even narrower than that of stainless steel, which is most likely due to different material properties, especially thermal conductivity, as it is about ten times higher for brass compared to stainless steel. The *S*_a_ value deviates slightly only at the maximum pulse repetition rate used, at which *E*_p_ is already so low that the ablation is basically no longer present (approximately 3.9% of *MRR*_max_) and thus the measurement itself is rather irrelevant.

The influence of different waveforms and consequently different pulse durations on the surface quality is negligible regardless of the studied material. As it follows from Figure 8b, the scatter of *S*_a_ is somewhat higher when stainless steel is involved. However, no clear correlation between the pulse length and the surface roughness can be found for any of the materials studied. Additional data on the conducted research, including 3D images of the samples, are available in Section S6.2 of the Supplementary Materials.

### 3.4. Influence of Scanner Parameters on MRR and S_z_

Based on the results of the first phase of this experimental study, which focused on the influence of laser processing parameters on the laser ablation, a set of parameters was selected for each studied material. These provide a favorable ratio between the *MRR* and the quality of the treated surface. They are listed in Table S2 of the Supplementary Materials and were further used to determine the effect of the scanner parameters (pulse-to-pulse overlap, line-to-line overlap, and scanning strategy) on *MRR* and surface roughness in the laser ablation process. Six different values (15–40 µm) of pulse spacing and scanning line separation were used, in other words, six different levels (0–62.5%) of pulse-to-pulse and line-to-line overlap as well as three different scanning strategies (0°, 0°/90° and 0°/45°/18°/72°). In order to optimize the evaluation of the results, the variable *S*_a_ was replaced by *S*_z_ (mean maximum surface depth) as a quality quantifier of the ablated surface, since previous results showed a minimal deviation from the linear relationship between them.

**Figure 9:**
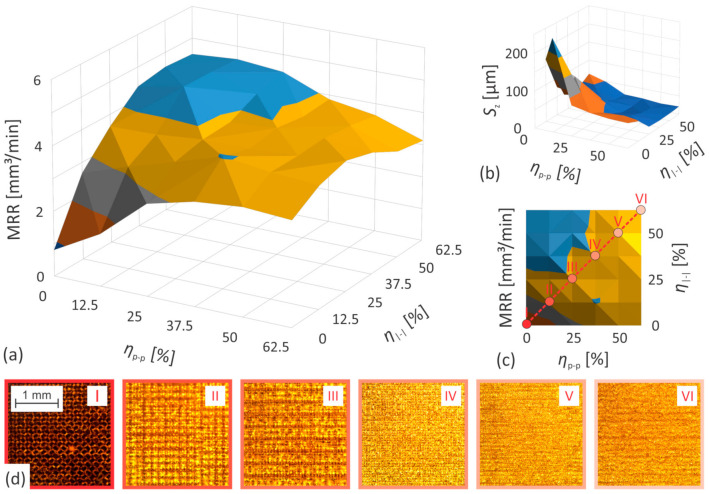
The evolution of (**a**,**c**) *MRR* and (**b**) surface roughness as a function of pulse-to-pulse and line-to-line overlap when processing brass CuZn37; (**d**) bottom of ablated areas acquired by an optical microscope using overlaps as they are marked with dots and Roman numerals on (**c**).

**Figure 10:**
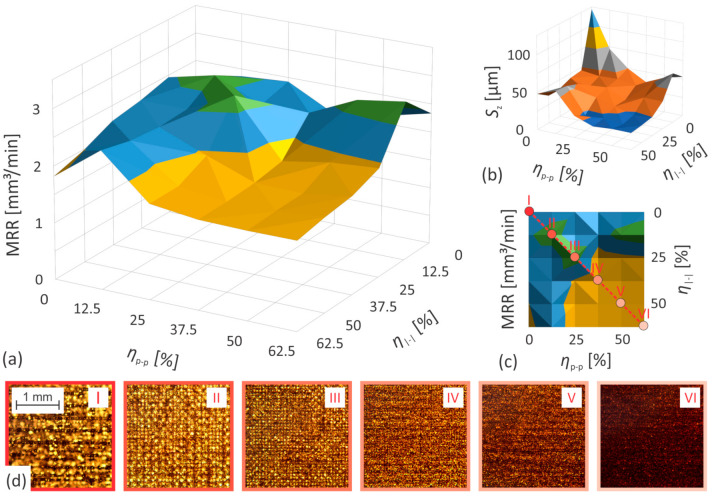
The evolution of (**a**,**c**) *MRR* and (**b**) surface roughness as a function of pulse-to-pulse and line-to-line overlap when processing steel AISI 316L; (**d**) bottom of ablated areas acquired by an optical microscope using overlaps as they are marked with dots and Roman numerals on (**c**).

Due to the sake of clarity, only the optimal results obtained with a 0°/90° scanning strategy are presented in Figure 9 and Figure 10. The other results are shown in Section S6.4, while details regarding the experiments are available in Section S5.3 of the Supplementary Materials. Figure 9 and Figure 10 show the *MRR* and surface roughness, as a function of pulse-to-pulse and line-to-line overlap. For the characteristic values of the variable parameters, visualization of the treated surface is also provided with images taken by an optical microscope (see inserted images in Figure 9d and Figure 10d). The processing parameters can be deduced from the matching labels (I–VI) in the upper right corners of the images (Figure 9d and Figure 10d) and the labels above the points in the 2D version of the *MRR* graph (Figure 9c and Figure 10c).

Regardless of the selected scanning strategy, the highest values of *MRR* are obtained at 25% and 37.5% of both, the pulse-to-pulse and the line-to-line overlap while processing stainless steel and brass, respectively. The *MRR* remains relatively constant if a small deviation from the optimal values of one of the overlaps is compensated by a corresponding compensation of the other overlap. However, this increases surface anisotropy (formation of directional roughness), and although the latter is not reflected in the surface quality quantifier (*S*_z_), it is usually undesirable and particularly noticeable when the scanning strategy consists of a small number of different scanning transitions (Figures S14e and S16e). There is also a considerable symmetry with respect to the axis formed by the matching combinations of the two studied overlaps (the dashed red lines in Figure 9c and Figure 10c). This confirms that the main influence of the overlaps is primarily related to the geometrical distribution of the laser pulses on the workpiece surface.

The roughness of the treated surface increases monotonically with a decrease in both levels of overlap, as shown in Figure 9b and Figure 10b. The trend is relatively moderate up to a certain value (approx. 37.5% and 25% of both the pulse-to-pulse and the line-to-line overlap while processing stainless steel and brass, respectively), after which it becomes much more pronounced. This tendency is quite similar to the behavior of the *MRR*, despite the fact that the maximum *S*_z_ values within the experimental range of variable parameters are not reached at all. With increasing overlap, the dimensions of the surface artifacts are visibly reduced and the structure itself becomes more and more homogeneous (Figure 9d and Figure 10d).

The influence of the scanning strategy on the quality of the treated surface is particularly noticeable at low levels of both overlaps, where a pronounced surface relief is generated, visually consistent with the scanning pattern (Figure 9d and Figure 10d). Namely, each pass over the surface leaves a certain structural orientation, which is further enhanced by the number of these passes, if the direction of the scanning remains constant. This leads to the formation of periodic structures, which usually manifest themselves as undesirable waviness of the surface. Their intensity decreases with the number of different transitions, as the previous relief orientation is partially erased by varying the direction of the scanning between individual scanning transitions. On the other hand, the influence of the scanning strategy on the material removal rate is considerably less noticeable. The achievable peak values of the *MRR* remain almost identical regardless of the selected scanning strategy. However, its dependence on both studied overlaps reduces with the increasing number of unique scanning transitions. By adding various transitions, additional spatial locations of laser pulses are provided and consequently the overlap is seemingly increased, thereby mitigating the *MRR* drop at *η* << 50% (see Figure 9, Figure 10 and Figures S14–S17 of the Supplementary Materials).

The process of laser ablation is therefore strongly related to the degree of both the pulse-to-pulse and the line-to-line overlap, on which the geometric distribution of the pulses on the workpiece surface indirectly depends. To further investigate these findings, we defined an additional so-called quality estimator (*QE*) which represents the relationship between the ablation rate and the final quality of the processed surface.

By taking into account that the *MRR* is defined as the volumetric amount of material removed in a unit of time [Equation (1)] and that the area and processing time were constant in all our experiments, the *QE* can be defined as a ratio between the ablation depth *h* and surface roughness *S_z_*:

(7) $$QE=\frac{h}{S_{z}}.$$

**Figure 11:**
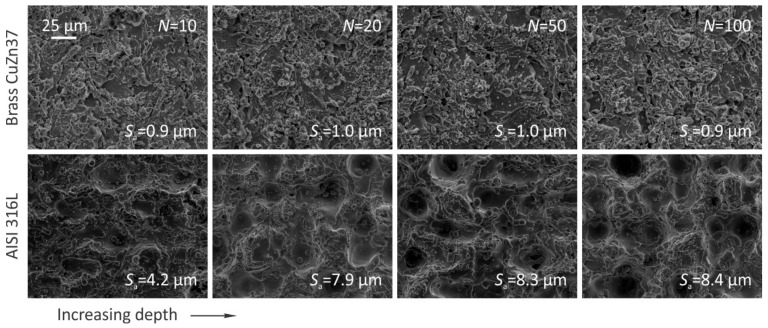
Bottom of the ablated areas as a function of scanning transitions.

It should be noted that the surface roughness is not entirely independent of the ablation depth. Namely, we have shown that for the range of parameters used in this study (Table S2 of the Supplementary Materials), the roughness of stainless steel increases with depth, whereas it is of depth independent when processing brass. The latter is evident from the SEM images of the ablated areas taken at different numbers of scanning transitions (Figure 11) and also from Figure S13 of the Supplementary Materials.

The differences between the materials are presumably a consequence of the different material properties, especially thermal conductivity, which is about 10 times higher for brass than for stainless steel. However, the main causes of this behavior are most likely the scanning strategy and the position of the laser beam focus itself. The scanning strategy defines the spatial distribution of the laser pulses at each scanning transition and is thus responsible for the potential amplification of the generated surface relief, whereas the position of the laser beam focus, which was stationary on the initial surface of the workpiece, affects the pulse fluence at the site of the light-matter interaction, as further discussed in Section S6.3.

**Figure 12:**
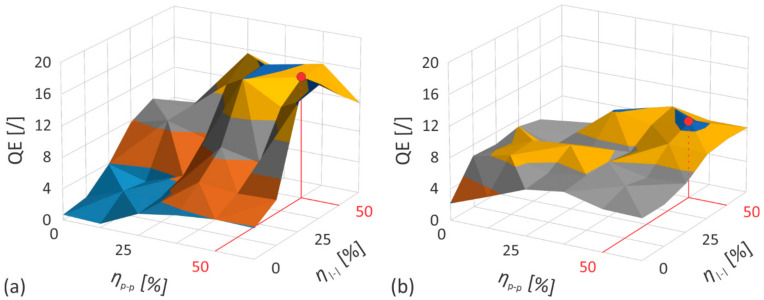
The evolution of *QE* as a function of pulse-to-pulse and line-to-line overlap when processing (**a**) brass CuZn37 and (**b**) steel AISI 316L, respectively.

Figure 12 shows the *QE* results obtained from the data, presented in Figure 9 and Figure 10. The existence of optimal parameters in terms of the achievable ratio between the material removal rate and the quality of the treated surface can be observed, with the maximum values of the process estimators being achieved at about 50% of both the pulse-to-pulse and the line-to-line overlap, independent of the studied material.

While the *QE* as a function of the pulse-to-pulse and the line-to-line overlap shows a certain continuity for both materials studied, the sensitivity of *QE* to any change in overlap is significantly higher for brass. Its peak value is therefore almost twice that of stainless steel, but on the other hand its minimum is also lower for the same experimental range of overlaps. The latter indicates that a more favorable ratios between the quality of the treated surface and the rate of material removal can be obtained when brass is ablated by the laser source with the same specifications. However, to achieve them, a detailed knowledge of the influences and dependencies between the influential parameters of the process is required.

## 4. Conclusions

We have investigated the influence of processing parameters on the ablation of AISI 316L steel and CuZn37 brass with a nanosecond, 1064-nm, Yb fiber laser. The effects of seven pulse durations from 70 ns to 240 ns, 14 pulse repetition rates between 20 kHz and 220 kHz, five steps between 0% and 62.5% of the pulse-to-pulse and the line-to-line overlap as well as three different scanning strategies on the material ablation rate (*MRR*) and the quality of the treated surface (measured by surface roughness *S*_a_) were studied. In addition, the effects of using different pulse fluences and different numbers of scanning transitions on the process outcomes were also investigated. We used a fast photographic technique to observe the processes leading to the macroscopic transformation of the surface, i.e., melting, vaporization, and plasma generation, which improved the understanding of the light-matter interaction at high repetition rates. From the presented results, we can draw the following general conclusions:

1. While the maximum achievable material removal rate *MRR* increases monotonically with the pulse length for both investigated materials, the influence of the pulse repetition rate is much more complex. *MRR* grows linearly up to the characteristic repetition rate *f*_0_ (where the pulse energy and the average power are both at maximal values) which is consistent with the increase in laser output power, and then the trend gradually reverses. The maximum *MRR* is reached at the pulse repetition rate that is higher or at most equal to *f*_0_. The determination of the exact value depends mainly on the correlation between the fluence of the laser pulses and their repetition rate and, thus, indirectly on a number of other processing parameters, including the pulse-to-pulse and line-to-line overlaps, the duration and shape of the pulses, and the scanning speed as well as on the material properties.
2. The quality of the treated surface is mainly influenced by the degree of overlap, whereby a larger overlap leads to a lower surface roughness, regardless of the material being processed. The effects of the pulse repetition rate are also noticeable, which determine the energy of the laser pulses, and of the scanning strategy, on which the amplitude of the waviness of the surface relief depends, while the influence of the pulse duration on surface roughness is practically negligible.
3. The process optimization indicates that while operating with laser processing parameters resulting in the highest *MRR*, the best achievable ratio between material removal rate and the quality of the treated surface is achieved at about 50% overlap of the laser pulses, regardless of the material being processed.

The presented results show the significance of the influence of numerous processing parameters on the laser ablation outcome. This study proves that by identifying these individual influences it is possible to improve the process from both a technological and physical point of view. As a result, more favorable ratios between the quality of the treated surface and the rate of material removal are achievable, which is essential for meeting the requirements for successful industrial implementation of the studied process. Since we used two metals with very different thermal conductivity, our conclusions about the influence of the processing parameters on the *MRR* and surface quality can be generalized to other metals. However, for different metals the variation of the optimal values of these parameters should be taken into account.

## Figures and Tables

**Table 1:** Physical properties of steel AISI 316L and brass CuZn37 [60,61,62].

Properties	Symbol	Unit	Steel AISI 316L	Brass CuZn37
Density	*ρ*	kg/m^3^	7900	8450
Melting point	*T*_m_	°C	1390	920
Boiling point	*T*_v_	°C	2800	1100
Latent heat of melting	*L*_m_	kJ/kg	290	168
Latent heat of boiling	*L*_v_	kJ/kg	6090	3680
Specific heat capacity	*c*_p_	kJ/kg K	470	377
Thermal conductivity (at 20 °C)	*κ*	W/mK	15	125

**Table 2:** Relevant parameters of the detection system.

System Characteristics	Photron
Frame rate [fps]	100k
Image size [pix]	640 × 280
Measuring area [mm]	12.8 × 5.6
Captured frames []	300
Color (gray) [bit]	16
Shutter time [µs]	8.4
Gamma correction []	1.5
System characteristics	Photron

## Data Availability

The data presented in this study are available on request from the corresponding author.

## Supplementary Materials

The following supporting information can be downloaded at: https://www.mdpi.com/article/10.3390/nano12020232/s1, Figure S1: The evolution of the pulse energy and the average power as a function of the pulse repetition rate; Figure S2: The pulse power as a function of time for (a) different pulse repetition rates (for the waveform 11) and (b) different waveforms (at the corresponding *f*_0_); Figure S3: Laser beam guidance across the surface of the workpiece; Figure S4: Peak pulse fluence as a function of the pulse repetition rate using (a) a 5 mm and (b) a 7.5 mm beam expander; Figure S5: Experimental strategy; Figure S6: Characteristic 3D measured profile of the surface of (a) the steel AISI 316L (*t*_p_ = 240 ns; *f* = 20 kHz) and (b) brass CuZn37 (*t*_p_ = 240 ns; *f* = 20 kHz) sample; Figure S7: Truncated 4-sided pyramid; Figure S8: Scanning strategies. (a) Transitions: 0°. (b) Transitions: 0°/90°. (c) Transitions: 0°/45°/18°/72°; Figure S9: (a) *MRPP* and (b) number of laser pulses per time unit (*NpT*) as a function of pulse fluence when processing steel AISI 316L; Figure S10: Energy efficiency of ablation of (a) brass and (b) stainless steel as a function of the pulse repetition rate using waveform 11 and two different laser system configurations; Figure S11: The influence of the laser processing parameters on the surface quality when processing brass CuZn37; Figure S12: The influence of the laser processing parameters on the surface quality when processing stainless steel AISI 316L; Figure S13: The influence of the ablation depth and processing atmosphere on the surface quality when processing (a) brass CuZn37 and (b) steel AISI 316L in air (the blue points) and argon (the red points) atmospheres; Figure S14: The evolution of (a,c) *MRR* and (b) surface roughness as a function of pulse-to-pulse and line-to-line overlap when processing brass CuZn37; scanning strategy: 0°; (d,e) bottom of ablated areas acquired by an optical microscope using overlaps as they are marked with dots on (c); Figure S15: The evolution of (a,c) *MRR* and (b) surface roughness as a function of pulse-to-pulse and line-to-line overlap when processing brass CuZn37; scanning strategy: 0°/45°/18°/72°; (d) bottom of ablated areas acquired by an optical microscope using overlaps as they are marked with dots on (c); Figure S16: The evolution of (a,c) *MRR* and (b) surface roughness as a function of pulse-to-pulse and line-to-line overlap when processing steel AISI 316L; scanning strategy: 0°; (d,e) bottom of ablated areas acquired by an optical microscope using overlaps as they are marked with dots on (c); Figure S17: The evolution of (a,c) *MRR* and (b) surface roughness as a function of pulse-to-pulse and line-to-line overlap when processing steel AISI 316L; scanning strategy: 0°/45°/18°/72°; (d) bottom of ablated areas acquired by an optical microscope using overlaps as they are marked with dots on (c); Table S1: The main characteristics of the used waveforms: *WF* is the number of the waveform, *E*_p,max_ stands for the maximum pulse energy, *t*_FWHM_ is the pulse duration at the full-width at half maximum, *t*_p,10_ is the pulse duration at 10% of the peak power, while *P*_max_ shows the peak power; Table S2: Laser processing parameters; steel AISI 316L; Table S3: Laser processing parameters; brass CuZn37; Table S4: Laser processing parameters used to determine the influence of scanner parameters on the ablation process; Table S5: Scanner parameters; steel AISI 316L; Table S6: Scanner parameters; brass CuZn37, Supplementary Materials.pdf.
