# Supplementary material for: The Influence of the Processing Parameters on the Laser-Ablation of Stainless Steel and Brass during the Engraving by Nanosecond Fiber Laser
Source: Nanomaterials (Basel). 2022 Jan 11;12(2):232. doi: 10.3390/nano12020232 (PMC8779350; doi:10.3390/nano12020232)
Supplement: Supplementary file 1 [file nanomaterials-12-00232-s001.zip › nanomaterials-1511297-supplementary.pdf]

Supplementary Materials

The Influence of the Processing Parameters on the Laser-Ablation of Stainless Steel and Brass during the Engraving by Nanosecond Fiber Laser

Luka Hribar, Peter Gregorčič *, Matej Senegačnik and Matija Jezeršek

Faculty of Mechanical Engineering, University of Ljubljana, Aškerčeva 6, 1000 Ljubljana, Slovenia; luka.hribar@fs.uni-lj.si (L.H.); matej.senegacnik@fs.uni-lj.si (M.S.); matija.jezersek@fs.uni-lj.si (M.J.)

* Correspondence: peter.gregorcic@fs.uni-lj.si; Tel.: +386-1477-1172

Table of Contents

S1 Fiber-laser characteristics

Fig. S1: The evolution of the pulse energy and the average power as a function of the pulse repetition rate.

Fig. S2: The pulse power as a function of time for (a) different pulse repetition rates (for the waveform 11) and (b) different waveforms (at the corresponding f_0).

Table S1: The main characteristics of the used waveforms: WF is the number of the waveform, $E_{p,max}$ stands for the maximum pulse energy, t_{FWHM} is the pulse duration at the full-width at half maximum, $t_{p,10}$ is the pulse duration at 10% of the peak power, while P_{max} shows the peak power.

S2 Experimental design

Fig. S3: Laser beam guidance across the surface of the workpiece.

Fig. S4: Peak pulse fluence as a function of the pulse repetition rate using (a) a 5 mm and (b) a 7.5 mm beam expander.

Fig. S5: Experimental strategy.

S3 Chemical composition of the material

S4 Measurements of the ablated volume

Fig. S6: Characteristic 3D measured profile of the surface of (a) the steel AISI 316L (t_p = 240 ns; f = 20 kHz) and (b) brass CuZn37 (t_p = 240 ns; f = 20 kHz) sample.

Fig. S7: Truncated 4-sided pyramid.

S5 Processing parameters

Table S2: Laser processing parameters; steel AISI 316L.

Table S3: Laser processing parameters; brass CuZn37.

Table S4: Laser processing parameters used to determine the influence of scanner parameters on the ablation process.

Fig. S8: Scanning strategies. (a) Transitions: 0°. (b) Transitions: 0°/90°. (c) Transitions: 0°/45°/18°/72°.

Table S5: Scanner parameters; steel AISI 316L.

Table S6: Scanner parameters; brass CuZn37.

S6 Results

Fig. S9: (a) MRPP and (b) number of laser pulses per time unit (NpT) as a function of pulse fluence when processing steel AISI 316L.

Fig. S10: Energy efficiency of ablation of (a) brass and (b) stainless steel as a function of the pulse repetition rate using waveform 11 and two different laser system configurations.

Fig. S11: The influence of the laser processing parameters on the surface quality when processing brass CuZn37

Fig. S12: The influence of the laser processing parameters on the surface quality when processing stainless steel AISI 316L.

Fig. S13: The influence of the ablation depth and processing atmosphere on the surface quality when processing (a) brass CuZn37 and (b) steel AISI 316L in air (the blue points) and argon (the red points) atmospheres.

Fig. S14: The evolution of (a,c) MRR and (b) surface roughness as a function of pulse-to-pulse and line-to-line overlap when processing brass CuZn37; scanning strategy: 0°; (d,e) bottom of ablated areas acquired by an optical microscope using overlaps as they are marked with dots on (c).

Fig. S15: The evolution of (a,c) MRR and (b) surface roughness as a function of pulse-to-pulse and line-to-line overlap when processing brass CuZn37; scanning strategy: 0°/45°/18°/72°; (d) bottom of ablated areas acquired by an optical microscope using overlaps as they are marked with dots on (c).

Fig. S16: The evolution of (a,c) MRR and (b) surface roughness as a function of pulse-to-pulse and line-to-line overlap when processing steel AISI 316L; scanning strategy: 0°; (d,e) bottom of ablated areas acquired by an optical microscope using overlaps as they are marked with dots on (c).

Fig. S17: The evolution of (a,c) MRR and (b) surface roughness as a function of pulse-to-pulse and line-to-line overlap when processing steel AISI 316L; scanning strategy: 0°/45°/18°/72°; (d) bottom of ablated areas acquired by an optical microscope using overlaps as they are marked with dots on (c).

S1 Fiber-laser characteristics

The pulse energy E_p and the average power P_{avg} as a function of the pulse repetition rate f for different waveforms is schematically presented in Fig. S1.

While each waveform has its own specific f_0, the general characteristics of the laser system remains unchanged regardless of the selected waveform. Up to f_0, the average power linearly increases with the pulse repetition rate while the pulse energy remains constant. The latter is intentionally limited to prevent any potential damage to the optical components of the laser source. When repetition rate reaches f_0, both the average power and the pulse energy are maximal. At pulse repetition rates above the f_0, the situation is reversed. The average power stays constant, while the pulse energy decreases according to Eq. (S1):

$$E_{\mathrm{p}} = \frac{P_{\mathrm{avg}}}{f} \tag{S1}$$

Table S1 shows the main characteristics of the waveforms that we used in this research. While all values can be found in the technical data provided by the manufacturer, we also measured them ourselves, thus taking into account possible losses in the experimental system. The pulse energy E_p and its duration t_p were simultaneously measured while applying individual waveforms using a calibrated energy meter (Ophir Optronics Inc., Israel, Smart head to USB interface with a pyroelectric sensor PE50BF-DIF-SH-U2) and a silicon photodiode (DET10A/M, manufactured by Thorlabs, 200 nm – 1100 nm). Assuming a linear response of the photodiode, the pulse power $P(t)$ is proportional to the measured voltage signal $U(t)$:

$$P(t) = CU(t) \tag{S2}$$

The proportionality constant C was determined from the obtained measurements since the pulse energy as a function of time is defined as:

$$E_p(t) = C\int_0^{t_p} U(t)\,dt \tag{S3}$$

The average power was experimentally measured by employing a power meter (Solo P/E, manufactured by Electro-Optics Inc., sensor head UP25N-100H-H9-D0) at the output of the scanning head.

The evolution of the pulse shape as a function of the pulse repetition rate and the selected waveform is presented in Fig. S2(a) and Fig. S2(b), respectively. For the sake of clarity, only the results for one selected waveform (WF 11) and for one set of repetition rates (f_0) are shown.

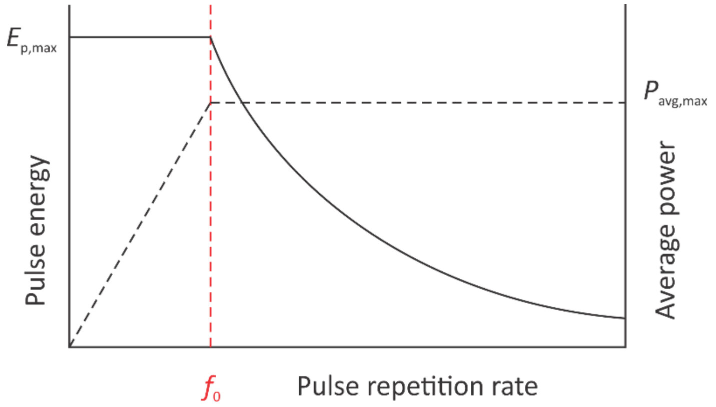

Figure S1. The evolution of the pulse energy and the average power as a function of the pulse repetition rate.

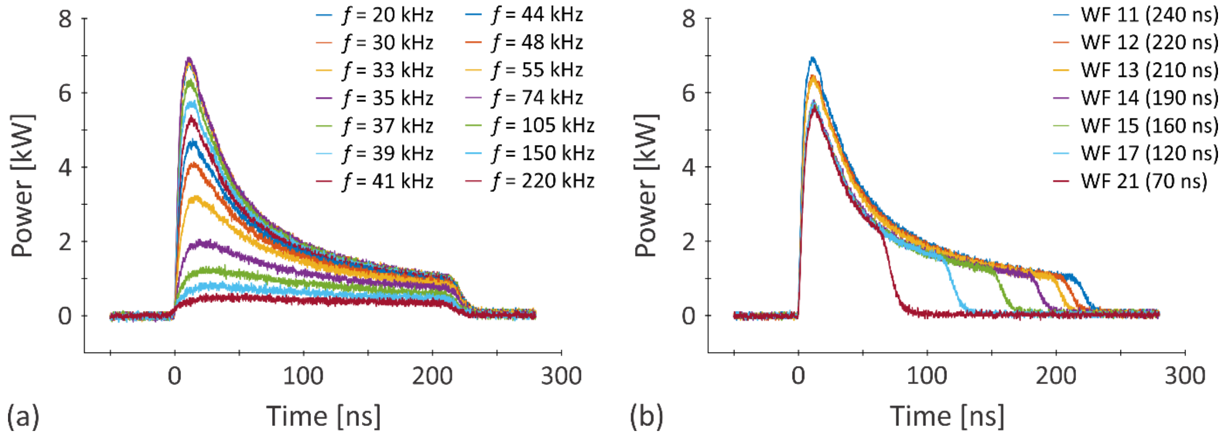

Figure S2. The pulse power as a function of time for (a) different pulse repetition rates (for the waveform 11) and (b) different waveforms (at the corresponding f_0).

Table S1: The main characteristics of the used waveforms: WF is the number of the waveform, $E_{p,max}$ stands for the maximum pulse energy, t_{FWHM} is the pulse duration at the full-width at half maximum, $t_{p,10}$ is the pulse duration at 10% of the peak power, while P_{max} shows the peak power.

WF	f_0 [kHz]	$E_{p,max}$ [mJ]	t_{FWHM}* [ns]	$t_{p,10}$* [ns]	P_{max}* [kW]
11	35	0.55	32	240	6.96
12	37	0.52	31	220	6.48
13	39	0.49	31	210	6.44
14	44	0.44	26	190	5.80
15	48	0.40	26	160	5.75
17	55	0.35	26	120	5.80
21	74	0.26	27	70	5.67

* Value measured at f_0.

S2 Experimental design

The pattern, consisting of parallel lines aligned in an arbitrary direction and having a constant spacing Δy, was chosen as the laser beam guiding trajectory to form a single scanning transition (Fig. S3). In order to optimize the processing time, the laser beam was alternately guided in both directions and the ablation was only interrupted at the transition to the new line (the dashed red lines in Fig. S3).

The study was conducted with two different configurations of the experimental system, using a 5 mm and a 7.5 mm beam expander, respectively. This directly affected the achievable laser light intensity as well as its fluence and, consequently, the ablation regime. Maximum fluence values as a function of the pulse repetition rate are presented in Fig. S4 for the two configurations used.

All experiments were performed with constant orientation of the workpiece since rolled metal sheets usually exhibit a certain structural orientation. Due to potential impact on laser-light absorption any significant heat accumulation in the material was also eliminated by alternately ablating the pockets at the distant areas of the workpiece (Fig. S5) and software implementation of a time delay (120 s) at each laser transition from one location to another (black arrows between the red squares in Fig. S5).

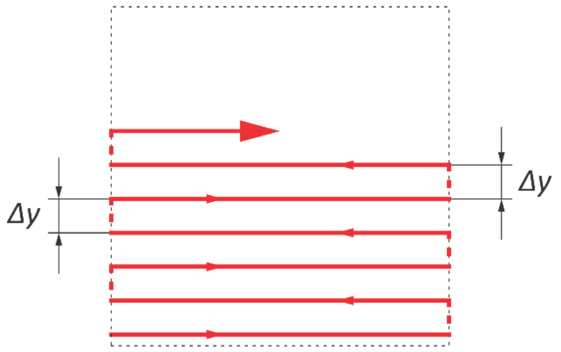

Figure S3. Laser beam guidance across the surface of the workpiece.

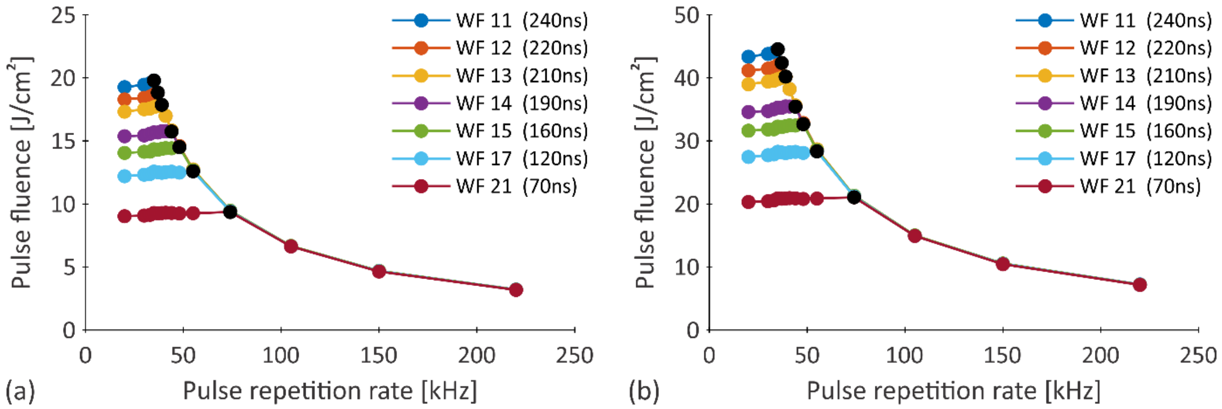

Figure S4. Peak pulse fluence as a function of the pulse repetition rate using (a) a 5 mm and (b) a 7.5 mm beam expander.

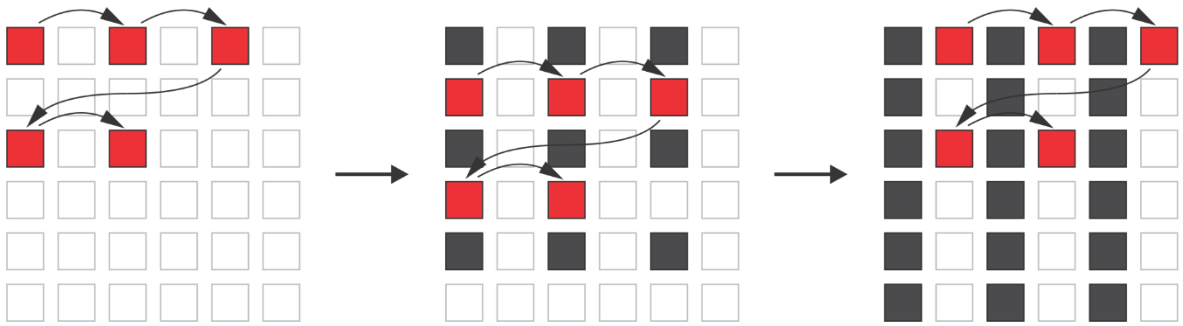

Figure S5. Experimental strategy.

S3 Chemical composition of the material

The chemical composition of the samples was analyzed using an X-ray fluorescence spectrometer XRF (Thermo Scientific Niton XL3t GOLDD+) and it is as follows (in wt. %):

- low-carbon stainless steel AISI 316L: Cr 16.9; Ni 10.1; Mo 2.1; Mn 1.9; Si 0.49; Cu 0.38; V 0.1; Fe the rest

- brass CuZn37: Zn 35.3; Cu the rest

S4 Measurements of the ablated volume

The ablated volume was measured on a few characteristic samples using a confocal optical microscope (Alicona InfiniteFocus). The device, based on a computer-controlled focal variation, captures a series of contrasting images from the observed layers of the sample and combines them into a three-dimensional model as shown in Fig. S6. Based on findings that are summarized in the section 2.3 of the main text, we decided to use a right 4-sided truncated pyramid (Fig. S7) to approximate the shape of the ablated pocket. The ablated volume V can, thus, be calculated as:

$$V = \frac{a^3}{6}\tan\alpha - \frac{1}{3}\left(a - \frac{2\,h}{\tan\varphi}\right)^2 \left(\frac{a}{2}\,h\right) \tag{S4}$$

where a is the outer dimension of the scanning field, h is the pocket depth and φ is the slope of the pocket walls. The latter was assessed to 64.5° based on preliminary tests, regardless of the processing material. An optical microscope with a displacement detection module was then used to measure the depths of the pockets, as already explained in the main text (section 2.3). To minimize the uncertainty of the results due to the potential surface waviness, the depth of each pocket was measured at four different locations and the average value was calculated as:

$$h_{\mathrm{avg}} = \frac{1}{4}\sum_{i=1}^{4} h_i \tag{S5}$$

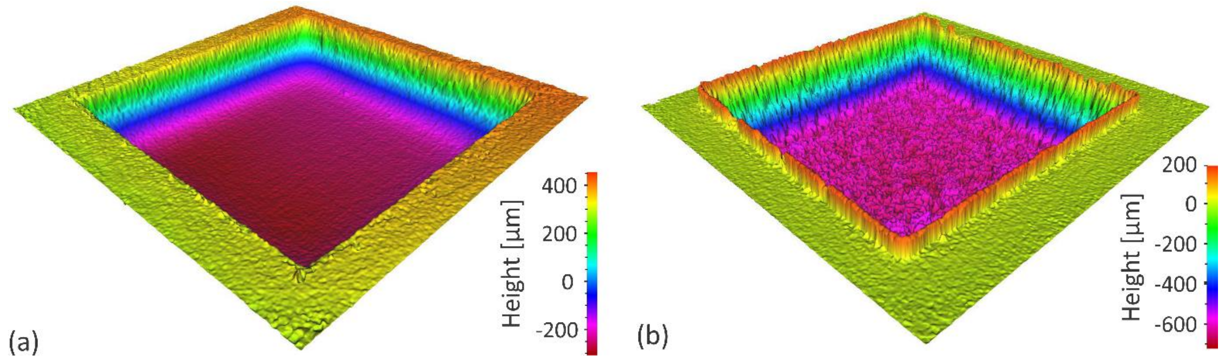

Figure S6. Characteristic 3D measured profile of the surface of (a) the steel AISI 316L (t_p = 240 ns; f = 20 kHz) and (b) brass CuZn37 (t_p = 240 ns; f = 20 kHz) sample.

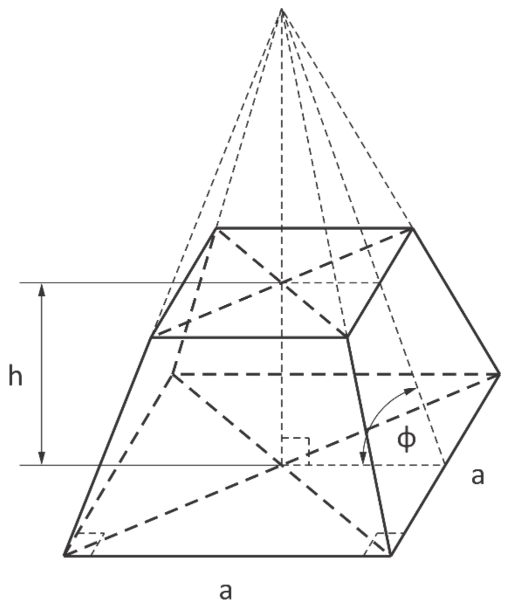

Figure S7. Truncated 4-sided pyramid.

S5 Processing parameters

The parameters used in this experimental study are presented in the following subsections.

S5.1 Laser processing parameters

Steel AISI 316L

The processing parameters for the ablation of steel AISI 316L are summarized in Table S2. The values of other influential parameters remained constant during the study:

- average power of the laser P_{avg} [W]: 18*
- vertical deviation of the surface from the beam waist z [mm]: 0
- scanning line separation Δy [µm]: 30
- scanning strategy: 0°/90°
- processing atmosphere: air

* relevant for pulse repetition rates higher than f_0.

After switching from lower to higher pulse fluences, only the number of scanning transitions had to be adjusted due to the increase in *MRR* so that the depth of markings would not exceed the thickness of the samples:

- number of scanning transitions *N*: 300 → 150

Table S2: Laser processing parameters; steel AISI 316L.

	**Pulse repetition rate *f* [kHz]**														
**t_p [ns]**	20	30	33	35	37	39	41		44	48	55	74	105	150	220	**WF**
240																11
220																12
210			Workpiece I, Workpiece III								Workpiece II, Workpiece IV					13
190																14
160																15
120																17
70																21
	600	900	990	1050	1110	1170	1230		1320	1440	1650	2220	3150	4500	6600	
	**Scanning speed *v* [mm/s]**															

Brass CuZn37

The processing parameters for the ablation of brass CuZn37 were slightly adjusted based on the intermediate results when changing from lower to higher pulse fluences. Although this directly affected the experimental results, the comparability of the process estimators was not compromised.

Experiments with a lower pulse fluences were carried out with the same set of parameters used in the study of stainless steel (Table S2) while the adjusted parameters used with higher pulse fluences can be found in Table S3. The values of majority of the influential parameters remained constant during the study:

- average power of the laser P_{avg} [W]: 18*
- vertical deviation of the surface from the beam waist z [mm]: 0
- scanning strategy: 0°/90°
- processing atmosphere: air

* relevant for pulse repetition rates higher than f_0.

Only the number of scanning transitions and spacing between scanning traces were adjusted after the change from lower to higher pulse fluences:

- number of scanning transitions N: 100 → 75
- scanning line separation Δy [µm]: 30 → 25

Table S3: Laser processing parameters; brass CuZn37.

	**Pulse repetition rate *f* [kHz]**															
**t_p [ns]**	20	30	33	35	37	39	41		44	48	55	74	105	150	220	**WF**
240																11
220																12
210			Workpiece III								Workpiece IV					13
190																14
160																15
120																17
70																21
	500	750	825	875	925	975	1025		1100	1200	1375	1850	2625	3750	5500	
	**Scanning speed *v* [mm/s]**															

S5.2 Ablation depth and processing atmosphere

Determination of the influence of ablation depth and processing atmosphere on the process outcome was performed by engraving pockets with a sequential increase in the number of scanning transitions (10, 20, 50, and 100) in two different working atmospheres, air and argon. The values of the other influential parameters that were constant during the study can be found below (AISI 316L steel or CuZn37 brass):

- pulse length t_p [ns]: 240 (WF 11)
- pulse repetition rate f [kHz]: 48 or 74
- average power of the laser P_{avg} [W]: 18*
- laser beam diameter on the lens d_l [mm]: 7.5
- pulse fluence [J/cm^2]: 32.6 or 21.3
- vertical deviation of the surface from the beam waist z [mm]: 0
- scanning strategy: 0°/90°
- scanning line separation Δy [µm]: 30 or 25
- scanning speed v [mm/s]: 1440 or 1850

* relevant for pulse repetition rates higher than f_0.

The working atmosphere was ensured by feeding the selected medium (argon or atmospheric air) into the processing chamber at a constant overpressure of 0.7 bar.

S5.3 Scanner parameters

The laser processing parameters that were selected on the basis of the previous findings and were further used to determine the influence of the scanner parameters on the ablation process are listed in Table S4. As already mentioned in the main text, three different scanning strategies (Fig. S8) and six different values of pulse spacing and scanning line separation were used.

The range of pulse spacing and scanning line separation in which the individual parameters were varied was determined by preliminary tests. Details for each material can be found in the following subsections.

The surface roughness parameter S_z, defined as the mean maximum surface depth, was calculated by measuring the vertical distance between the deepest valley and the highest peak at five different locations on the surface, which were then averaged. Measurements were conducted on the optical microscope with a displacement detection module (described in section 2.3) using the optical focusing method.

Table S4: Laser processing parameters used to determine the influence of scanner parameters on the ablation process.

	**Steel AISI 316L**	**Brass CuZn37**
***Pulse length t_p* [ns]**	240 (WF 11)	240 (WF 11)
***Pulse repetition rate f* [kHz]**	48	74

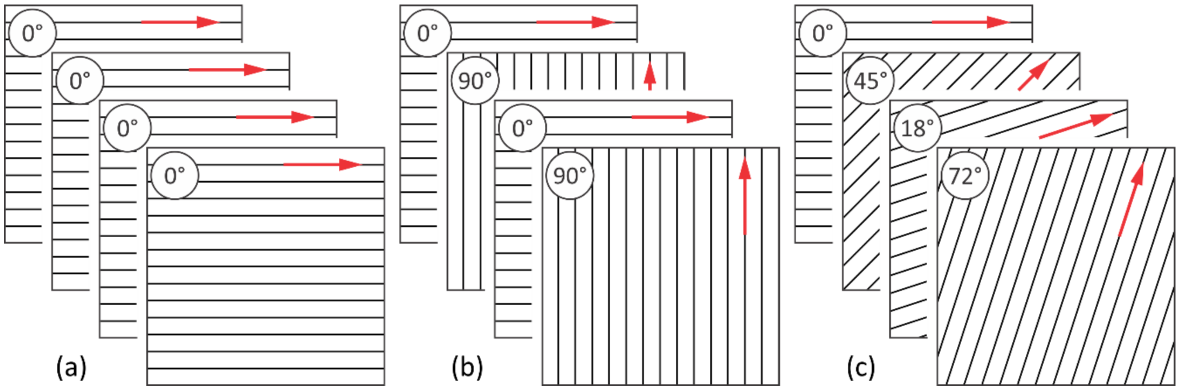

Figure S8. Scanning strategies. (a) Transitions: 0°. (b) Transitions: 0°/90°. (c) Transitions: 0°/45°/18°/72°.

Steel AISI 316L

Initial tests, primarily aimed at optimizing the rate of material subtraction, have shown that the highest *MRR* values are obtained when both the pulse-to-pulse overlap $\eta_{p\text{-}p}$ and the line-to-line overlap $\eta_{l\text{-}l}$ are in the range of 25% (gray colored rectangle in Table S5). Based on these results, the range of the variable parameters was centered as presented in Table S5.

Table S5: Scanner parameters; steel AISI 316L.

	Δy						
Δx	40	35	30	25	20	15	**$\eta_{l\text{-}l}$ [%]**
40							0
35							12.5
30							25
25							37.5
20							50
15							62.5
**$\eta_{p\text{-}p}$ [%]**	0	12.5	25	37.5	50	62.5	
	1920	1680	1440	1200	960	720	
	***v* [mm/s]**						

Brass CuZn37

The highest *MRR* we achieved at about 37.5% line-to-line and pulse-to-pulse overlaps when ablating brass (gray colored rectangle in Table S6). Since, compared to the study performed on stainless steel, the maximum remained in the middle of the range of variable parameters, the latter were not changed, only some indirectly dependent parameters were adjusted.

Table S6: Scanner parameters; brass CuZn37.

	Δy						
Δx	40	35	30	25	20	15	$\eta_{l\text{-}l}$ [%]
40							0
35							12.5
30							25
25							37.5
20							50
15							62.5
$\eta_{p\text{-}p}$ [%]	0	12.5	25	37.5	50	62.5	
	2960	2590	2220	1850	1480	1110	
	v [mm/s]						

S5.4 Plasma characterization

Processing parameters used in the plasma characterization study that are not listed in the main text (section 2.4) can be found below (AISI 316L steel or CuZn37 brass):

- pulse length t_p [ns]: 240 (WF 11)
- average power of the laser P_{avg} [W]: 18*
- laser beam diameter on the lens d_l [mm]: 7.5
- vertical deviation of the surface from the beam waist z [mm]: 0
- scanning strategy: 0°/90°
- scanning line separation Δy [µm]: 30 or 25
- processing atmosphere: air

* relevant for pulse repetition rates higher than f_0.

S6 Results

S6.1 Influence of laser processing parameters on *MRR*

Figure S9 shows the same set of graphs as presented in Fig. 4 in the main text, with this one being associated with the study of stainless steel. Conclusions regarding the interdependence between the fluence of the laser pulses and the pulse repetition rate are identical to those presented in section 3.1 of the main text.

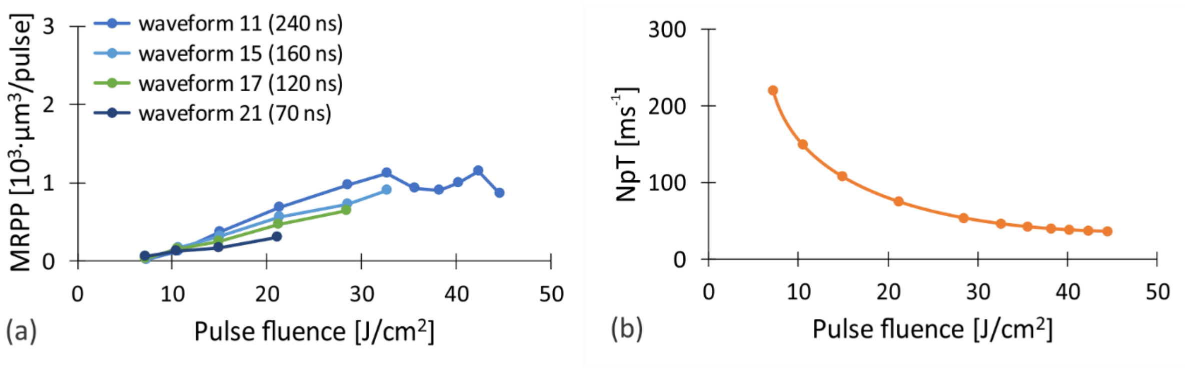

Figure S9. (a) MRPP and (b) number of laser pulses per time unit (*NpT*) as a function of pulse fluence when processing steel AISI 316L.

S6.2 Influence of laser processing parameters on S_a

As part of the study regarding the influence of the pulse repetition rate on the surface roughness, the results were also presented in the form of energy efficiency η_E. Figure S10 shows the calculated η_E of stainless steel and brass ablation as a function of the repetition rate using WF 11 and two different laser system configurations (7.5 mm and 5 mm beam expander), i.e. two different sets of pulse fluences. Energy efficiency is defined as the ratio between the theoretical amount of energy required to evaporate the ablated material Q and the actual energy input E_{tot}:

$$\eta_E = \frac{Q}{E_{tot}} = \frac{m\, q_v}{E_{tot}} \quad \text{(S6)}$$

In this case, m is the mass of the ablated material and q_v is the heat required to evaporate 1 kg of this specific material, defined as:

$$q_v = c_p\,(T_v - T_0) + L_m + L_v \quad \text{(S7)}$$

where c_p is the specific heat capacity, T_v is the boiling point, T_0 is the temperature of the workpiece before machining, L_m is the latent heat of melting and L_v is the latent heat of boiling. Equation S6

can be further transformed to contain the variables measured during this study, expressing the energy efficiency as the ratio of the actual (*MRR*) and the theoretically possible amount of material removed in a given time unit (MRR_t), the latter being defined as:

$$MRR_t = \frac{V_t}{t} = \frac{P_{\text{avg}}}{\rho\, q_{\text{v}}} \tag{S8}$$

V_t is the volume of the material that can theoretically be removed in time t with the input energy E_{tot}. The energy efficiency is thus defined as follows:

$$\eta_{\text{E}} = \frac{MRR}{MRR_{\text{t}}} = MRR \left(\frac{P_{\text{avg}}}{\rho\, q_v}\right)^{-1} \tag{S9}$$

The material properties required to calculate the selected quantity are listed in the main text (Table 1).

Details regarding the experimental study designed to determine the influence of the processing parameters on the laser ablation are evident from Fig. S11 and Fig. S12. Above the visual display of the samples, surfaces produced using different pulse repetition rates and the waveform 11 (t_p = 240 ns), captured with a Leitz optical microscope with 100x magnification are shown. Their variable process parameters can be deduced from the matching borders that are defined by different shades of red. For a few characteristic surfaces ($f << f_0$, $f \approx f_0$ and $f >> f_0$), their 3D reconstruction generated by the Alicona InfiniteFocus confocal light microscope is also presented. Below the samples, surfaces fabricated using different waveforms with corresponding repetition rates, which provide the maximum *MRR*, are shown in a similar way. The borders from which the values of the variable processing parameters can be deduced are colored in matching shades of yellow while the 3D surface reconstruction is shown for three waveforms with significantly different t_p (210, 160 and 70 ns).

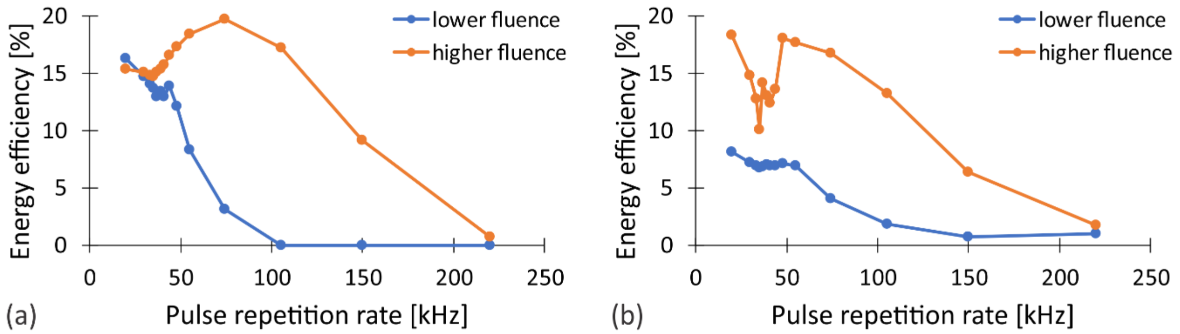

Figure S10. Energy efficiency of ablation of (a) brass and (b) stainless steel as a function of the pulse repetition rate using waveform 11 and two different laser system configurations.

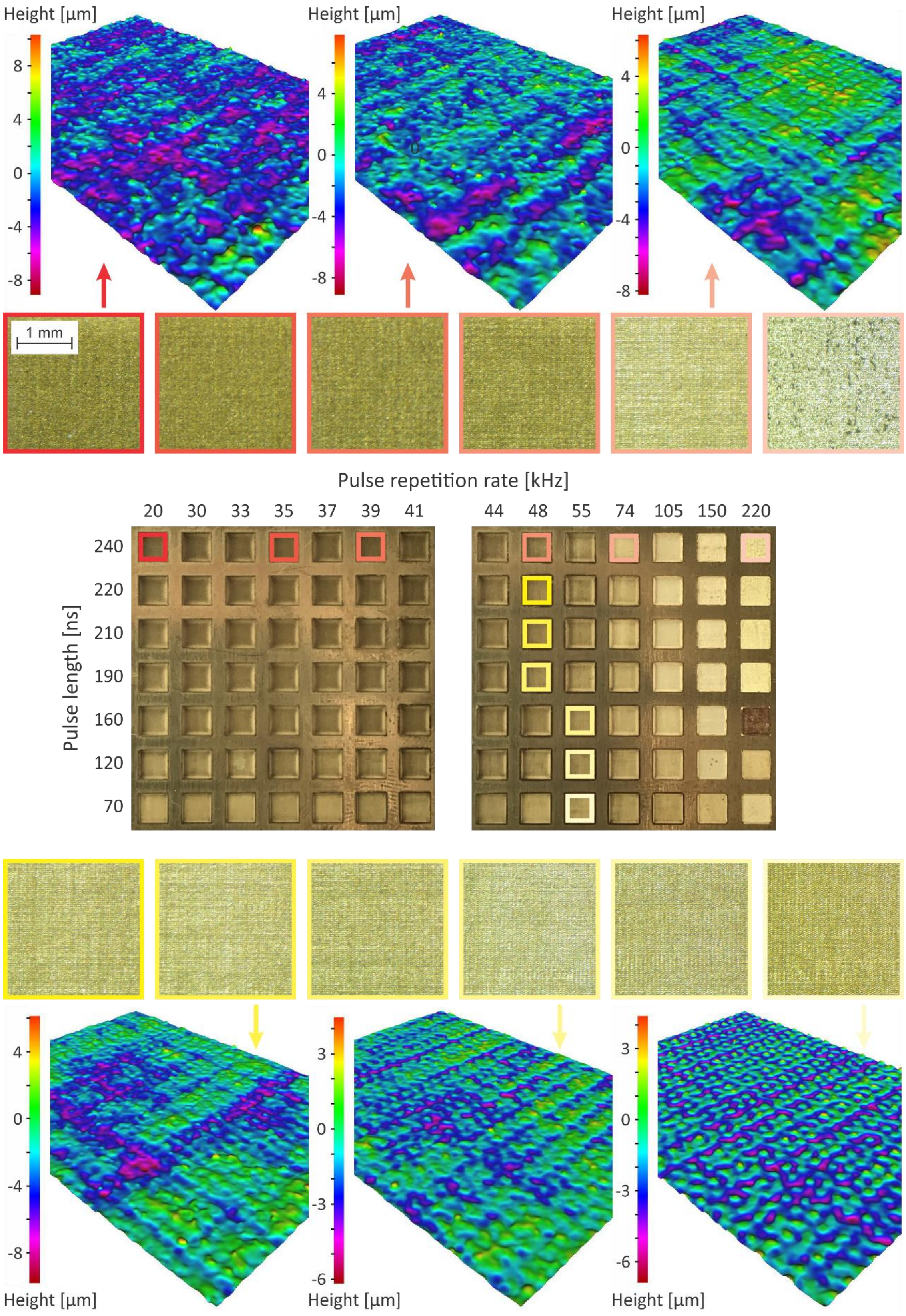

Figure S11. The influence of the laser processing parameters on the surface quality when processing brass CuZn37.

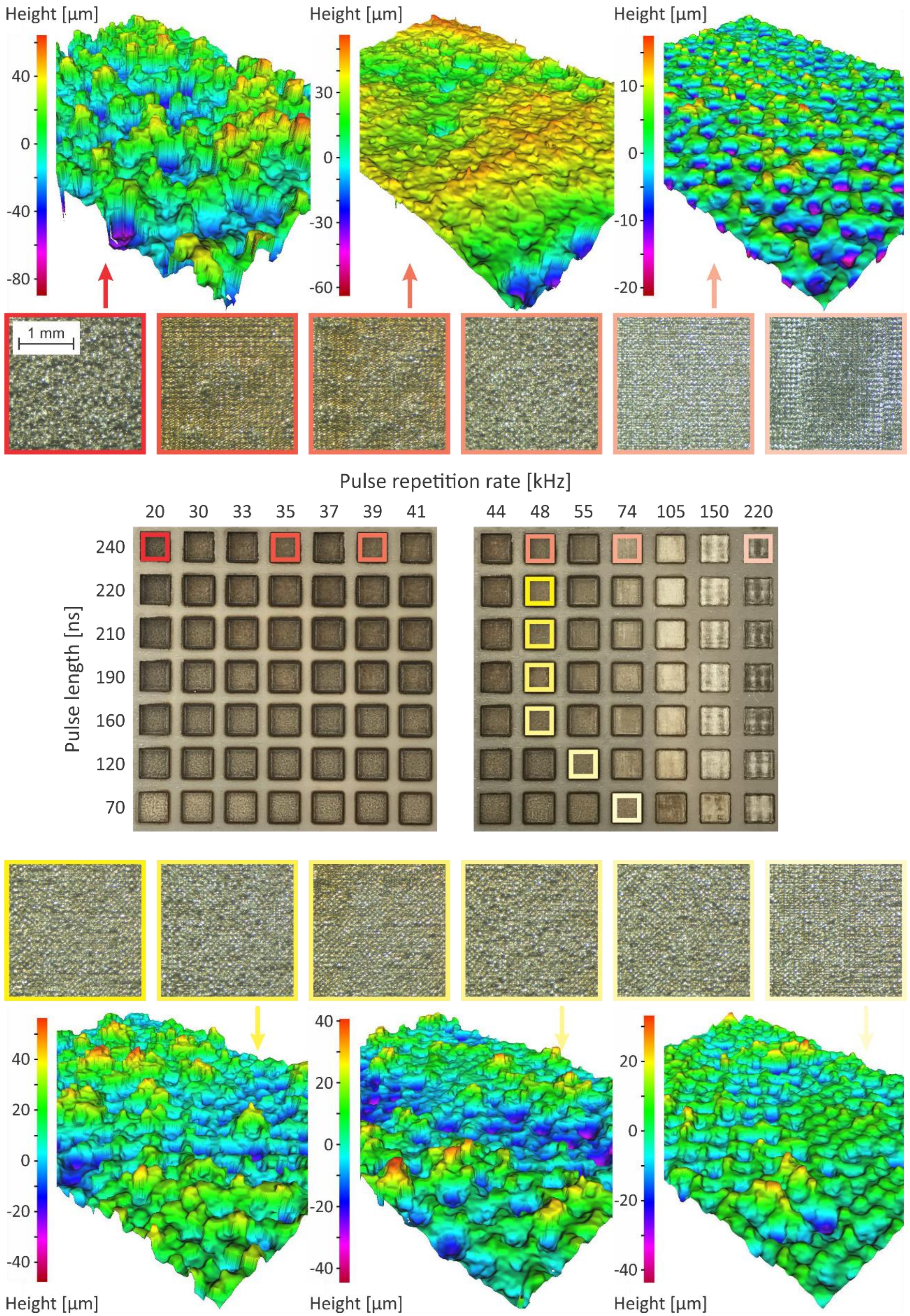

Figure S12. The influence of the laser processing parameters on the surface quality when processing stainless steel AISI 316L.

S6.3 Influence of scanning transitions and processing atmosphere on *MRR* and S_a

The experimental results of the study on the influence of the number of transitions and the processing atmosphere on the laser ablation are shown in Fig. S13.

The main conclusion that can be drawn from Fig. S13 is that the relationship between the number of scanning transitions and the achievable depth is distinctly linear for both materials studied, regardless of the process atmosphere used. The *MRR* is thus independent on the depth, but its value decreases significantly after the transition from air to inert gas (Ar) with a drop of approximately 50% and 75%, for stainless steel and brass, respectively.

The influence of the studied parameters on S_a is a bit more complex. S_a increases monotonically with depth when stainless steel is ablated in atmospheric air. In contrast, when brass is ablated, S_a is independent of depth. The main reason for this behavior is most likely the stationary position of the laser beam focus on the initial surface of the workpiece and the resulting defocusing of the light due to the increasing depth of the ablation volume. The differences between the materials, on the other hand, are presumably a consequence of the different material properties, especially the thermal conductivity, which is about 10 times higher for brass than for stainless steel. The opposite is true for the ablation in an inert atmosphere, where S_a of the stainless steel decreases and S_a of brass increases with depth. Further research is required to explain this process dynamics. However, the findings to date already show the potential to improve the quality of the treated surface by implementing various polishing phases in the ablation process.

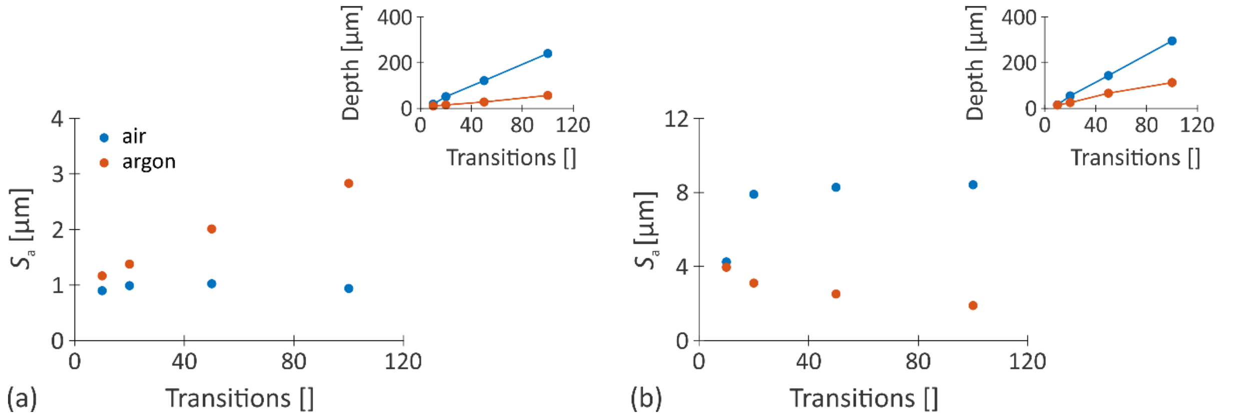

Figure S13. The influence of the ablation depth and processing atmosphere on the surface quality when processing (a) brass CuZn37 and (b) steel AISI 316L in air (the blue points) and argon (the red points) atmospheres.

S6.4 Influence of scanner parameters on *MRR* and S_z

Figures S14-S17 present additional experimental results related to the study of the influence of scanner parameters on *MRR* and S_z in the laser ablation process. The information providing method is identical to that already described in the main text, and the results are consistent with the findings already presented in section 3.3 of the main text.

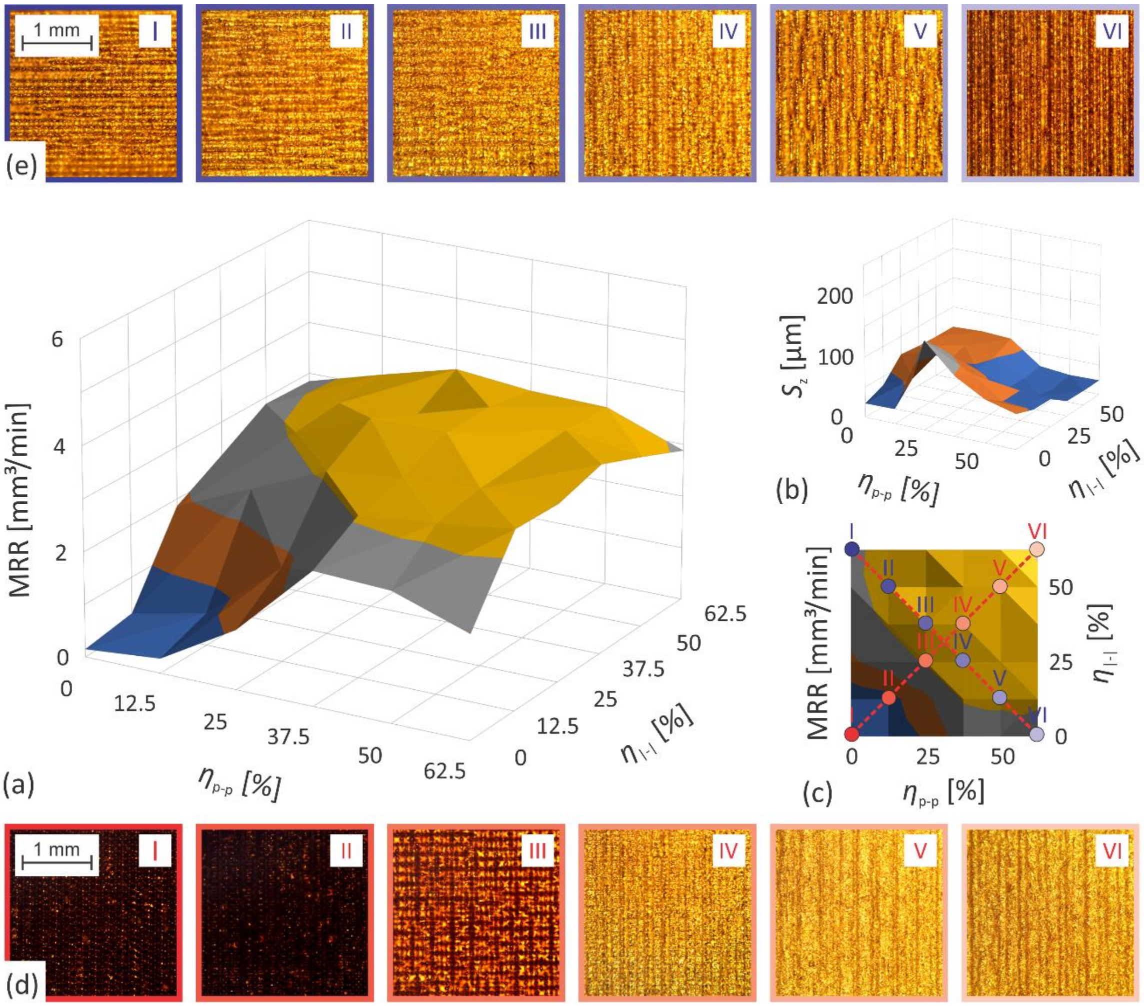

Figure S14. The evolution of (a,c) *MRR* and (b) surface roughness as a function of pulse-to-pulse and line-to-line overlap when processing brass CuZn37; scanning strategy: 0°; (d,e) bottom of ablated areas acquired by an optical microscope using overlaps as they are marked with dots on (c).

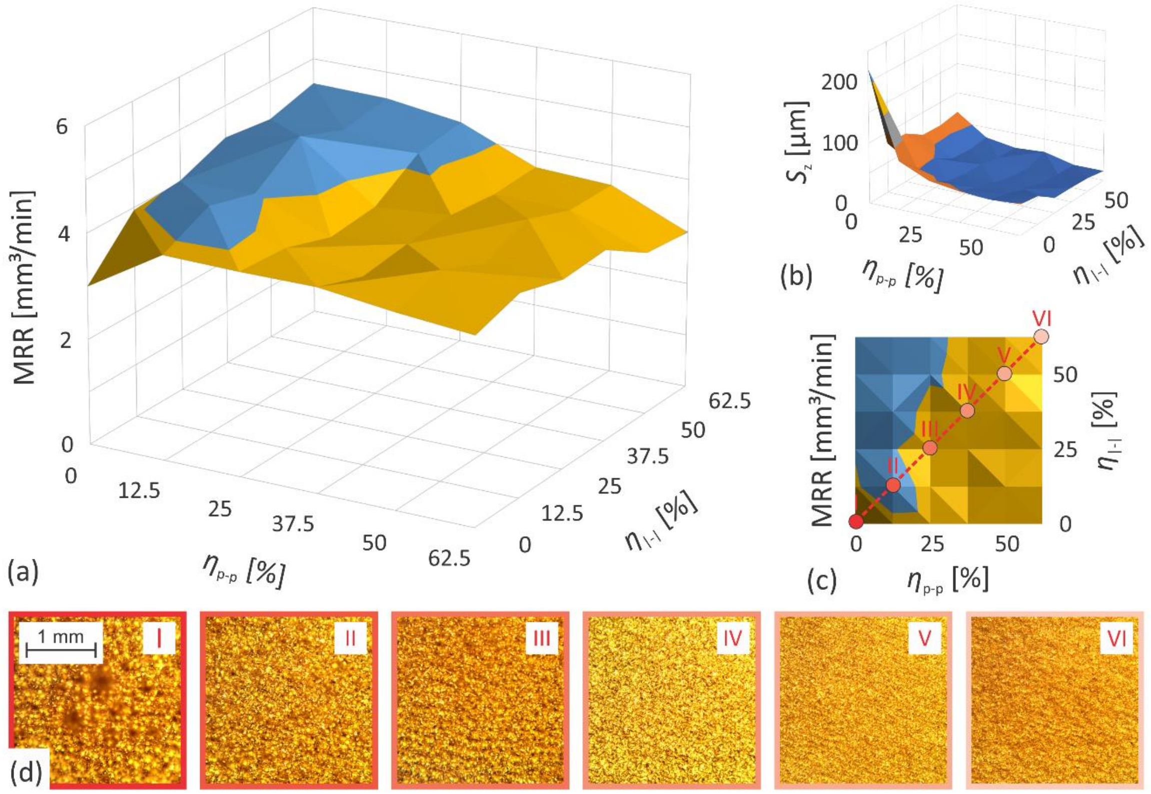

Figure S15. The evolution of (a,c) *MRR* and (b) surface roughness as a function of pulse-to-pulse and line-to-line overlap when processing brass CuZn37; scanning strategy: 0°/45°/18°/72°; (d) bottom of ablated areas acquired by an optical microscope using overlaps as they are marked with dots on (c).

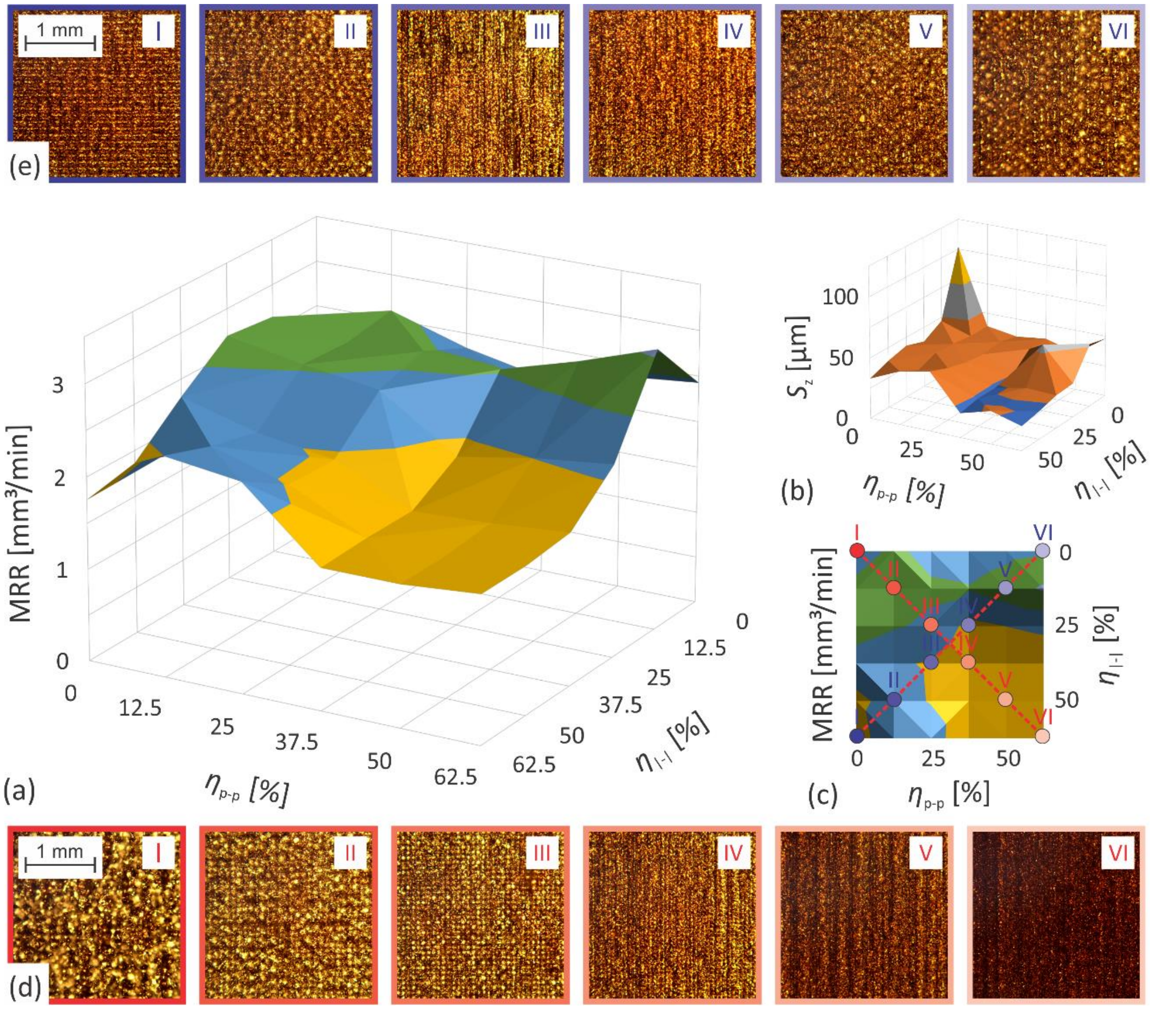

Figure S16. The evolution of (a,c) *MRR* and (b) surface roughness as a function of pulse-to-pulse and line-to-line overlap when processing steel AISI 316L; scanning strategy: 0°; (d,e) bottom of ablated areas acquired by an optical microscope using overlaps as they are marked with dots on (c).

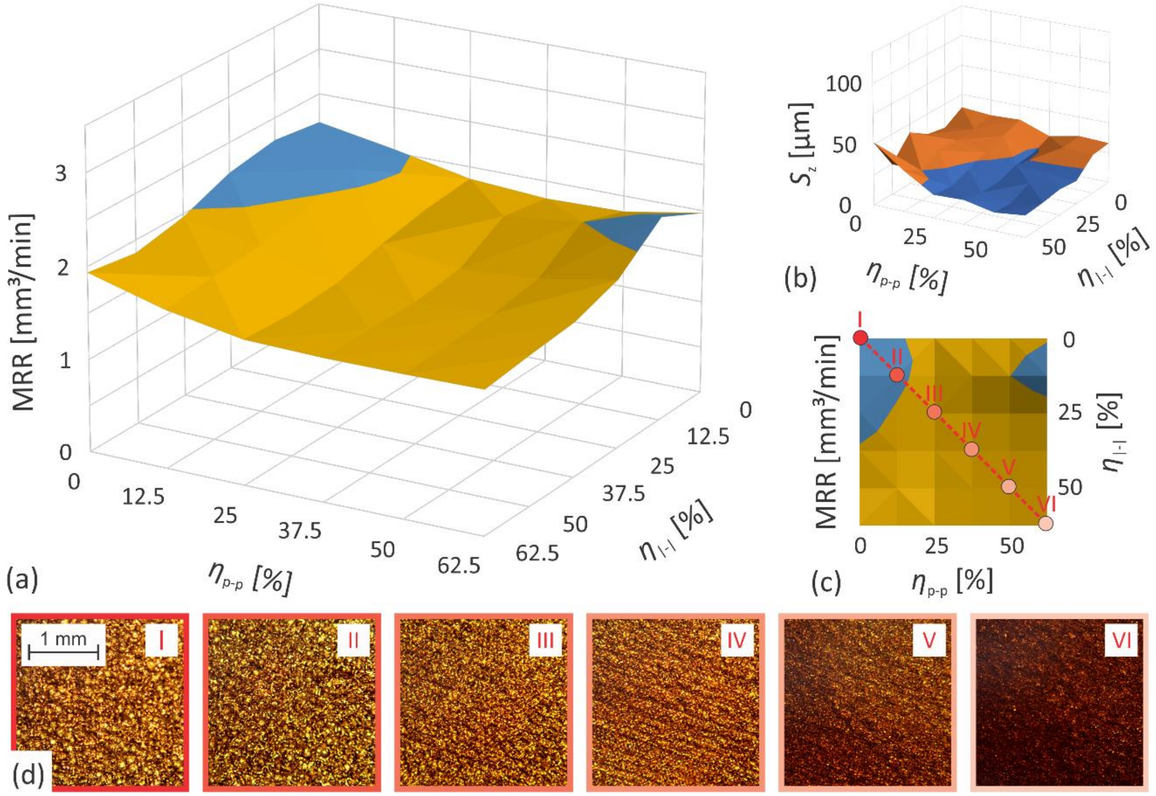

Figure S17. The evolution of (a,c) *MRR* and (b) surface roughness as a function of pulse-to-pulse and line-to-line overlap when processing steel AISI 316L; scanning strategy: 0°/45°/18°/72°; (d) bottom of ablated areas acquired by an optical microscope using overlaps as they are marked with dots on (c).
